# Multi-edge NEXAFS study of non-fullerene acceptors: electronic structure and molecular orientation supported by simulations of NEXAFS angular dependence

**DOI:** 10.1039/d6ra03796h

**Published:** 2026-07-03

**Authors:** Beatriz Molinaro Guerra, Marcin Zając, Andreas Opitz, Cleber F. N. Marchiori, Maria Luiza M. Rocco

**Affiliations:** a Institute of Chemistry, Federal University of Rio de Janeiro (UFRJ) Rio de Janeiro RJ Brazil luiza@iq.ufrj.br; b National Synchrotron Radiation Centre SOLARIS, Jagiellonian University Kraków Poland; c Institut für Physik, Humboldt-Universität zu Berlin Berlin Germany; d Department of Engineering and Physics, Karlstad University Karlstad Sweden cleber.marchiori@kau.se

## Abstract

Near-edge X-ray absorption fine structure (NEXAFS) spectroscopy is a powerful tool to probe the electronic structure of organic semiconductors. However, the interpretation of core-level excitations in chemically complex systems remains challenging. Here, we present a combined experimental and theoretical study of the carbon, nitrogen, and oxygen K-edges NEXAFS spectra of two non-fullerene electron acceptors widely used in organic photovoltaics: ITIC and IDTBR. The simulated angular-dependent NEXAFS spectra consistently reproduce the experimental data for all edges, enabling a detailed assignment of the main spectral features in terms of specific core-level excitations, variation of peak intensities, and final-state symmetries. The experimental dichroic ratios for ITIC indicate a higher degree of face-on organization at the surface compared to the bulk. For IDTBR, differences between surface- and bulk-sensitive measurements reveal a more complex behavior, where the observed trends depend on the specific absorption edge, reflecting variations in the localization of the excited states and the degree of molecular torsion. Theoretical dichroic ratios become higher when the in-plane components of the transition dipole vector show a magnitude comparable to its perpendicular component. These results further support the tendency of ITIC toward a face-on orientation at the surface, while indicating a higher degree of structural disorder in IDTBR. Additionally, a comparative assessment of exchange–correlation functionals was performed by evaluating M06 against B3LYP and PBE, showing that M06 outperforms the others, especially in the carbon K-edge. By providing a unified interpretation of experimental NEXAFS spectra across multiple edges, this work offers new insights into the electronic structure of these acceptors and establishes a robust framework for analyzing core-level excitations in complex organic materials.

## Abstract 2

Multi-edge NEXAFS combined with TDDFT provides a unified interpretation of electronic structure, molecular orientation, and core-level excitations in non-fullerene acceptors.

## Graphical abstract

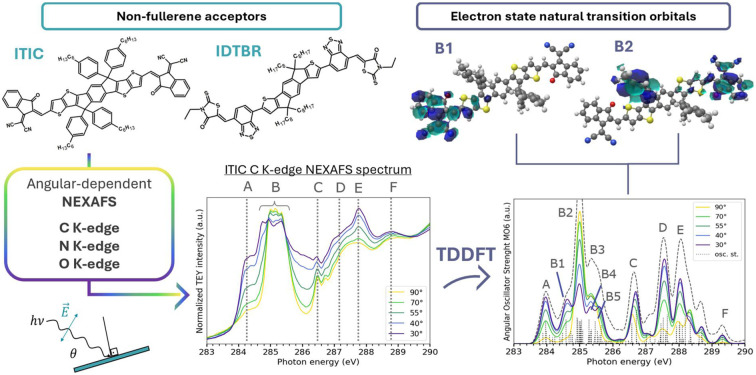

## 1. Introduction

**Fig. 1:**
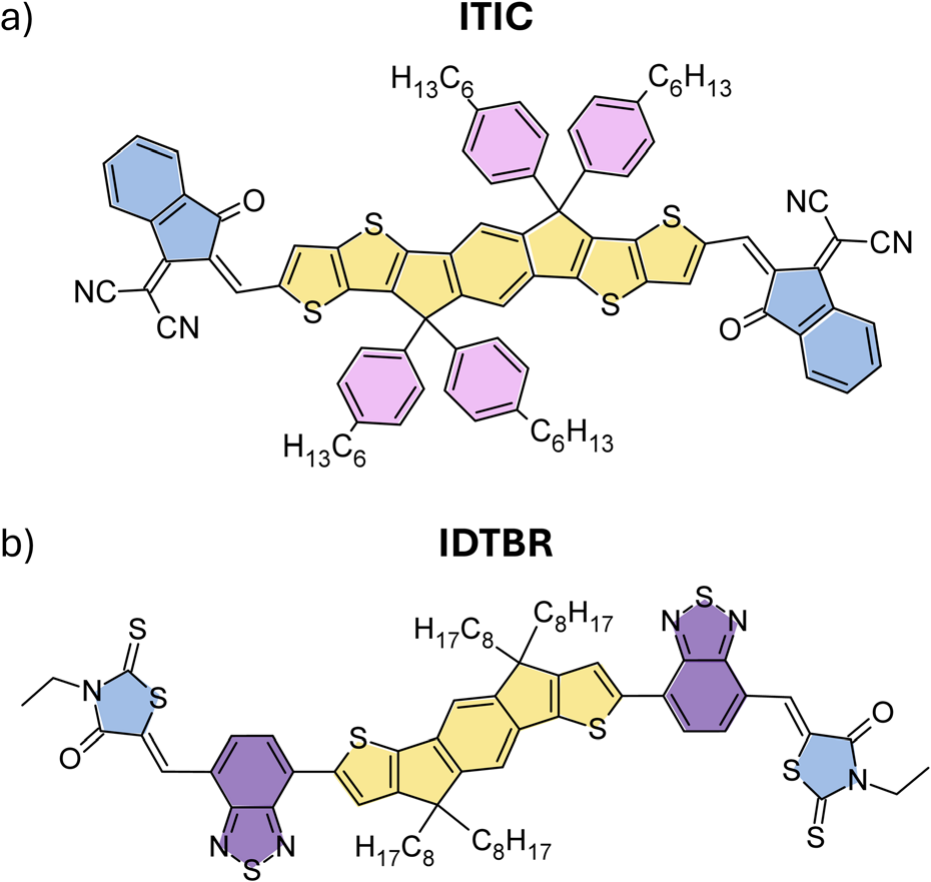
Molecular structures representations of (a) ITIC and (b) IDTBR Acceptor units rings are colored with blue, donor units rings with yellow, side chains rings with pink, and π spacer rings (extended acceptor unit for this case) with purple.

Organic semiconductors have become an established strategic material for optoelectronic devices due to their low weight, mechanical flexibility, semitransparency, and compatibility with solution-based processing.^1–4^ In organic photovoltaics (OPVs), the active layer is commonly formed by blending an electron donor and an electron acceptor, and solution-processing them into thin films.^1–4^ Non-fullerene acceptors (NFAs) are a class of organic molecules that follow a molecular design strategy already widely used for donor materials: intercalating two or more units with different electronic properties.^5^ This molecular design enables the control of the energy gap and HOMO/LUMO energy levels, and their predominantly planar conjugated backbones, combined with alkyl side chains flanking the core, promote solubility and improved mechanical properties as well as morphological stability.^2–5^ A decisive breakthrough occurred in 2015 with the introduction of 3,9-bis(2-methylene-(3-(1,1-dicyanomethylene)-indanone))-5,5,11,11-tetrakis(4-hexylphenyl)-dithieno[2,3-*d*:2′,3′-*d*′]-*s*-indaceno[1,2-*b*:5,6-*b*′]dithiophene, known as ITIC (Fig. 1a), a small-molecule acceptor with an A–D–A (acceptor–donor–acceptor) architecture, whose performance surpassed that of the fullerene-based systems and firmly established NFAs as viable alternatives to former applied electron acceptors.^5–7^ Further modularity can also be achieved by inserting π-conjugated spacer units between the central core and terminal groups. In this context, (5*Z*,5′*Z*)-5,5'-((7,7'-(4,4,9,9-tetraoctyl-4,9-dihydro-*s*-indaceno[1,2-*b*:5,6-*b*′]dithiophene-2,7-diyl)bis(benzo[*c*][1,2,5]thiadiazole-7,4-diyl))bis(methanylylidene))bis(3-ethyl-2-thioxothiazolidin-4-one), known as IDTBR or *o*-IDTBR (Fig. 1b) represents a prominent example of an A–A′–D–A′–A architecture, incorporating a benzothiadiazole which has acceptor characteristics.^5,8–10^ Due to their relatively narrow energy gaps, materials like ITIC and IDTBR can act simultaneously as light absorbers and charge-transporters, contributing to the photo-induced charge photogeneration.^5–10^

As the electronic performance of these materials is strongly correlated with their molecular organization in thin films,^10,11^ studies with experimental techniques sensitive to both electronic structure and molecular orientation are essential. Near-edge X-ray absorption fine structure (NEXAFS) spectroscopy is particularly well suited for such a purpose. The intensity of core-level electronic transitions depends on the relative orientation between the electric field vector of the incident linearly polarized X-ray radiation and the symmetry of the molecular orbitals involved. By probing specific absorption edges, NEXAFS provides element and orbital selective information, enabling direct insight into electronic structure and molecular orientation in organic thin films.^12–20^

Precisely because this technique is very sensitive to the sample morphology and the chemical environment of the atom whose edge is being probed, the complete interpretation of NEXAFS spectra for large and complex conjugated organic molecules comprising multiple chemical environments remains challenging. The building-block approach is often insufficient, not only due to the limited availability of experimental data of small fragments, but also because of the substantial changes in the electronic structure when isolated molecular fragments are compared with the full conjugated molecule.^21^ In such cases, theoretical methods become indispensable for the reliable assignment of electronic transitions.

Density Functional Theory (DFT) has long been a central tool for describing the electronic and spectroscopic properties of organic materials.^16,22–29^ For example, time-dependent DFT (TDDFT) NEXAFS simulations have been performed to assess the effect of oxygen adsorption and oxidation on the oxygen K-edge spectra of fullerene-based organic semiconductors,^28^ to better understand the resonances in the nitrogen K-edge spectra of Y-series non-fullerene acceptors,^16^ and to study the adsorption of small organic radicals on metal surfaces.^27^ Although other works have proposed alternative theoretical methodologies for simulating core excitations in conjugated organic molecules,^23,26^ the standard DFT approach remains the most widely used method within the experimental spectroscopy community. In the theoretical field, some works aimed to refine functionals specifically for NEXAFS simulations, such as the Short-Range Correction functionals, or use a very high-exchange and range-separated functionals, such as BHHLYP, CAM-B3LYP, e ωB97X-D. They were tested for small organic molecules and showed good agreement.^30,31^ Franco and co-workers^29^ showed that for the large and conjugated non-fullerene acceptors ITIC and Y5 [commonly labeled bis(4-(di-*p*-tolylamino)phenyl)phenylphosphine oxide], the impact of a given exchange-correlation functional can be either positive or negative depending on the specific characteristics of each molecule, and on the electronic property being analyzed. This work highlights the importance of exploring more than one functional, even when the systems under investigation are similar in nature and have already been studied using functionals at the same theory level.

Another interesting and useful feature to explore in this kind of calculation is to predict the angular dependence of the transition intensities. The NEXAFS spectra for semiconducting polymers were theoretically studied by Su *et al.*^24^ using the excited electron and core-hole approach based on constrained-occupancy DFT. Among other discussions, like how the number of repeat units and the side chain lengths affect the simulated spectrum, they show that angular-dependent simulations can accurately reproduce experimental spectra and enable a reliable determination of molecular orientation in semiconducting polymer thin films, even in partially disordered systems. The interpretation of experimental spectra becomes more challenging for materials with significant conformational twisting and spectral overlap of transitions with opposite angular behavior, but they use this approach to enlighten these characteristics, highlighting the need for theoretical support.

In this work, we investigated the electronic structure and molecular orientation of two representative non-fullerene electron acceptors, ITIC and IDTBR, using NEXAFS at the carbon, nitrogen, and oxygen K-edges. Spectra were acquired in total electron yield (TEY) and fluorescence yield (FY) modes, providing complementary surface and bulk sensitive information, respectively. The results revealed that ITIC has a higher face-on organization, especially on the surface. Although IDTBR showed a preference for face-on orientation, it remains mostly disordered, particularly at the surface. TDDFT calculations are used to assign the dominant contributions underlying the main spectral features, unveiling their chemical origin. Additionally, a computational analysis of the angular dependence was employed by simulating the orientation of the linear-polarized X-ray electric field and applying it to the calculated vertical transitions in order to evaluate intensity variations associated with changes in molecular orientation. This framework adds insightful information to the calculated spectra, making it easier to assign the final-state symmetry. This comparative theoretical and experimental approach provides for a more robust interpretation of NEXAFS spectra of complex organic semiconductors.

## 2. Experimental section

### 2.1 Materials

ITIC (99%), IDTBR (99%), and chlorobenzene (99.9%) were purchased from Sigma-Aldrich and used as received. ITO-coated glass substrates were obtained from Kurt J. Lesker Company.

### 2.2 Sample preparation

Solutions of ITIC and IDTBR were prepared in chlorobenzene at a concentration of 20 mg mL^−1^ and stirred for 24 h before deposition. Thin films were deposited under ambient conditions by spin coating 65 µL of solution onto ITO/glass substrates (1.2 cm × 1.2 cm) at 2800 rpm for 30 s. After deposition, the films were thermally annealed on a hot plate at 150 °C for 15 min.

### 2.3 NEXAFS acquisition and data processing

NEXAFS measurements were carried out at the PIRX beamline at the SOLARIS National Synchrotron Radiation Center (Krakow, Poland).^32^ PIRX is a bending-magnet beamline optimized for light elements, covering absorption edges from 100 to 2000 eV. The X-ray beam was linearly polarized to probe molecular orientation. In previous work in this beamline,^16^ the degree of polarization was reported as (87 ± 4)%. The angular spectra were acquired at incidence angles of 90°, 70°, 55°, 40°, and 30° relative to the sample surface. Signals were collected simultaneously in total electron yield mode (TEY), measured by the sample drain current, and fluorescence yield mode (FY), recorded using a silicon drift detector (SDD, Amptek). Data treatment was carried out using PyMca 5.6.7.^33^ Savitzky–Golay smoothing was applied when required. Pre-edge ranges for the C, N, and O K-edges were 276–281 eV, 390–397 eV, and 520–528 eV, respectively, while post-edge ranges were 315–345 eV, 415–425 eV, and 560–572 eV. The dichroic ratio (*D*) in TEY mode was calculated using a relation between the extreme angles' intensities (*I*) in the peak maximum, where *D* = (*I*_90_ − *I*_30_)/(*I*_90_ + *I*_30_). In FY mode, spectra at 90° cannot be acquired due to experimental constraints in the beamline; therefore, the 70° spectra were used for the dichroic analysis.

### 2.4 Computational details

To assess the minimum theoretical level required for a reliable spectral description, DFT and TDDFT calculations were performed by ORCA^34^ 5.0.4 using three exchange-correlation functionals in a progressive level of Hartree–Fock (HF) exchange correction: PBE [Generalized Gradient Approximation – GGA, with no HF exchange], B3LYP (hybrid with 20% HF exchange), and M06 (hybrid *meta*-GGA with 27% HF exchange). The choice of functionals was guided by previous theoretical benchmarks,^26^ prioritizing methods that provide good agreement with experimental optical and fundamental gaps within their respective theory levels, such as M06, as well as their availability in ORCA and their use in the literature, like B3LYP and PBE.^19–21,23^ Since we are most interested in the 1s → π* region of the absorption spectrum, because that is the part of the spectra that gives most chemical and morphological information in studies about organic semiconductors thin films, the original alkyl side chains of the NFAs were replaced with methyl groups to reduce computational cost. As this approximation would already be included, we chose a simpler Gaussian basis set 6-311G** to be used in all stages of the calculation, as a basis set that included diffuse functions would be more relevant for higher energy transitions near or after the ionization potential, which is not the central objective of the work. We perform a geometry optimization for each functional, allowing full degree of freedom in all bonds. Then, the optimized structures were used to simulate the absorption spectra in carbon, nitrogen, and oxygen K-edges in their respective functional (see SI for more DFT/TDDFT details). Alternative conformations were not investigated due to the scope of the study and the associated computational cost. Solvent effects were included using the conductor-like polarizable continuum model with chlorobenzene (*ε* = 5.6968) as the solvent at all stages of the calculations, to mimic the dielectric constant of the molecular environment in the film. The natural transition orbitals (NTO) were generated using Avogadro, with an isovalue of 0.015 for the final-state orbitals associated with the carbon and nitrogen spectra and 0.010 for those associated with the oxygen spectra. Spectral convolution and visualization were done using Python 3.12.12. Each vertical electronic transition, obtained from the transition electric dipole moments, was convoluted with a Gaussian function *y* = *A* exp[–(*x* − *µ*)^2^/(2*σ*^2^)], where *A* corresponds to the oscillator strength (theoretical transition intensity), *µ* to the excitation energy, and *σ* to the spectral broadening. The *σ* values were fixed at 0.1 eV for the C K-edge, 0.3 eV for the N K-edge, and 0.5 eV for the O K-edge for all molecules and functionals. These values were selected empirically through visual inspection, with the sole criterion of providing a good representation of the experimental spectrum and a clear visualization of the spectral features. To simulate the angular dependence, the molecular *Z*-axis was defined as perpendicular to the conjugated backbone (Fig. S1), and orthogonal projections were computed between the transition dipole components along the *X*, *Y*, and *Z* molecular axes and a unit vector simulating the experimental electric field orientation at incidence angles of 90°, 70°, 55°, 40°, and 30°. The resulting values were multiplied by the corresponding oscillator strengths, and angular-dependent spectra were obtained from Gaussian convolution (see SI for more details about the assignment and the data treatment).

## 3. Results and discussion

In the following results, the nature of the main resonances that contribute most to the peaks will be discussed in terms of the NTOs representing their initial and final states, as well as the orientation tendency of the molecules. An edge-on orientation corresponds to molecules whose molecular planes are perpendicular to the substrate, whereas a face-on orientation indicates that the molecular planes are parallel to it. In terms of the dichroic ratio described here, values of −1 ≤ *D* < 0 indicate a preference for face-on alignment, 0 < *D* ≤ 1 correspond to an edge-on preference, and *D* ≅ 0 reflects the absence of preferential orientation. The face-on configuration is generally considered favorable for OPVs, as charge transport in such architectures typically occurs perpendicular to the substrate, for instance, from anode to cathode in the devices.^35,36^

### 3.1 ITIC

#### 3.1.1 Carbon

**Fig. 2:**
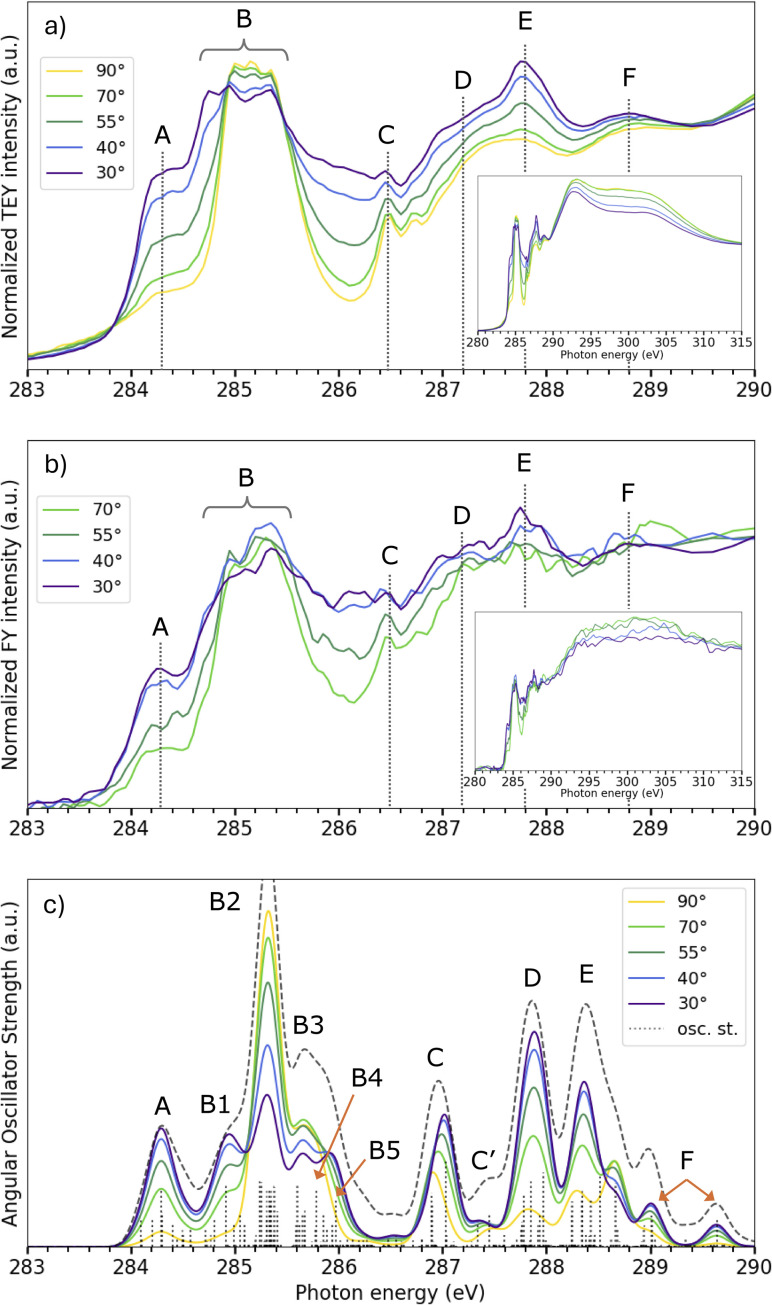
Experimental carbon K-edge NEXAFS in (a) TEY and (b) FY modes, and (c) calculated carbon K-edge NEXAFS obtained from TD-DFT calculation at M06/6-311G** theory level for ITIC.

Fig. 2 shows the experimental angular dependence of the carbon K-edge NEXAFS spectra of ITIC. The overall spectral profiles obtained in TEY (Fig. 2a), and FY (Fig. 2b) modes are nevertheless very similar.

The region between 284 and 290 eV corresponds to transitions 1s → π* and exhibits six main features located at 284.3 eV (A), 284.7–285.6 eV (B), 286.5 eV (C), 287.2 eV (D), 287.8 eV (E), and 288.8 eV (F). The dichroic ratios for the first π* transition are *D* = −0.44 in TEY mode and *D* = −0.32 in FY mode. These values point to a preferential face-on orientation at the surface and in the bulk of the film. However, one should note that the values of the dichroic ratios are low, especially in FY mode, and that the expected *D* to clearly infer a face-on orientation should be at least −0.5, preferentially closer to −1. Therefore, to a more precise assessment of molecular orientation, the dichroism ratio will also be calculated for the other edges and discussed together in the general discussion session.

Above 290 eV, the spectrum is dominated by 1s → σ* transitions, whose intensity is maximal at normal incidence as expected in a face-on orientation. But unlike what is typically observed for the π* region in this alignment, not all features increase in intensity as the incidence angle approaches 30°. While most peaks show an intensity enhancement towards 30°, especially pronounced for A and E, part of the transitions contributing to the broad peak B decrease, which initially suggests σ*-like character. To understand the origin of this unusual behavior, we need theoretical support. Overall, the M06 performance for all edges was better among the functionals, so all theoretical spectra presented in the text will be in this level of theory. The comparison between functionals will be developed in the general discussion session.

Fig. 2c shows the simulated carbon K-edge spectrum with the M06 functional (for other functionals, see Fig. S2). The dashed line corresponds to the Gaussian convolution of the oscillator strengths, and its shape is kind of strange in principle, especially for the simulated peaks B. However, when the simulated electric field is applied, the calculated transition intensities are strongly affected. Since the molecular backbone in the simulation is oriented parallel to the *XY* plane, representing an artificial substrate, and the simulated angles correspond to the experimental beam incidences, the resulting angular-dependent spectrum reproduces a face-on spectrum in agreement with experiment. Now, the five peaks convoluted in experimental peak B can be well described, as well as the others. Since peak B has the most non-straight forward analysis, it will be done after the theoretical analysis of the other resonances.

**Fig. 3:**
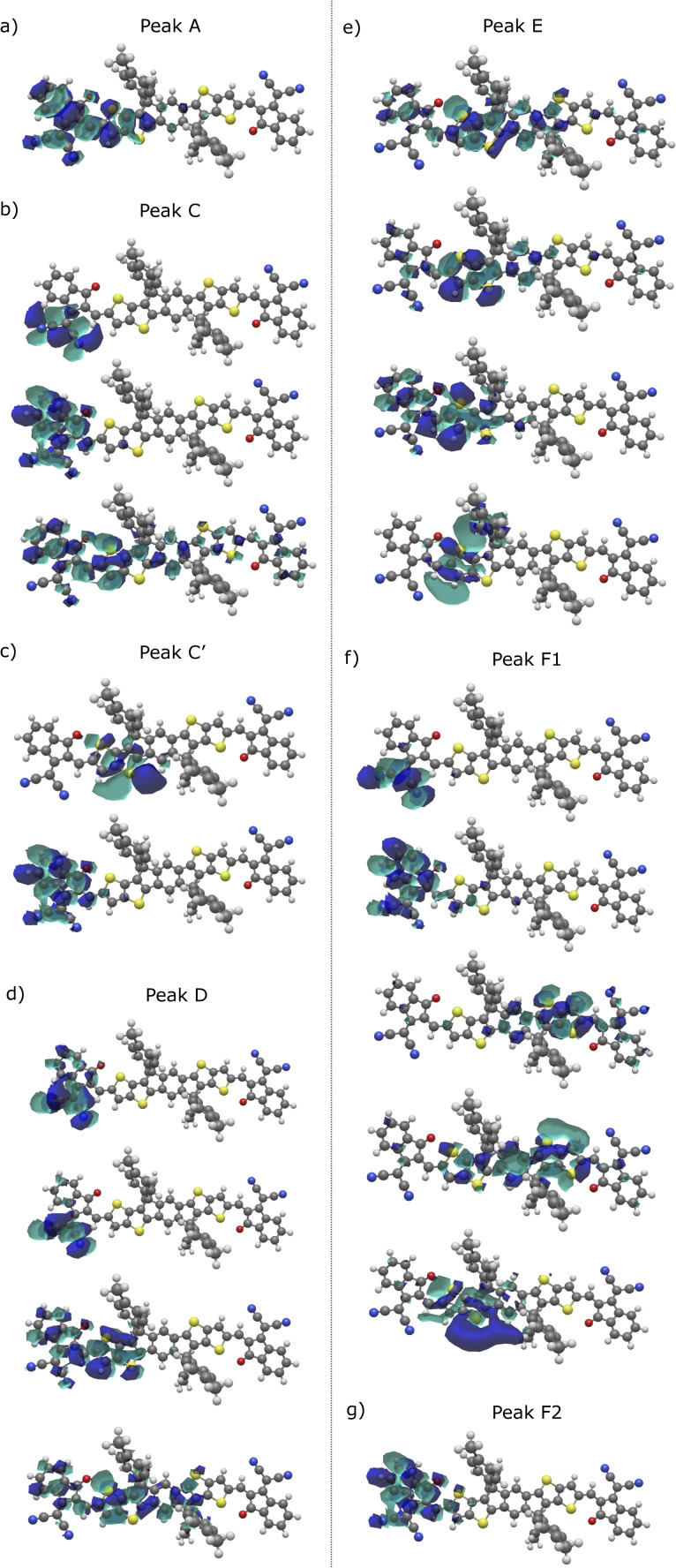
Final states natural transition orbitals (isovalue = 0.015) for the most representative transitions for peaks (a) A, (b) C, (c) C′, (d) D, (e) E, (f) F1, and (g) F2 in carbon K-edge NEXAFS spectra of ITIC simulated with M06/6-31G** level. Carbon atoms are colored in grey, hydrogen atoms in white, nitrogen atoms in blue, oxygen atoms in red, and sulfur atoms in yellow.

Peak A arises from a 1s electron excitation of the carbon atom linking the acceptor unit (indanone) to the thiophene ring. This transition leads to a state localized over the acceptor group and the thieno[3,2-*b*]thiophene core (Fig. 3a), whose spatial density distribution closely resembles that of the LUMO and LUMO + 1. Peak E originates from a few excitations in which the 1s electrons from the carbon in the C–S bonds are promoted to unoccupied orbitals mostly confined to the same thiophene unit (Fig. 3e). The favorable overlap between initial and final states, combined with their predominant π* character, accounts for the marked increase in intensity of peaks A and E in the experimental spectra as the beam incidence becomes more grazing.

Peak C is composed of two different transitions whose energies are sufficiently close to overlap. Its calculated energy is approximately 0.5 eV higher than that observed experimentally, which induces a shift to higher energy transition for the remaining spectral features. The first contribution originates from excitation of 1s electrons from the carbon in the C≡N bonds into a π* orbital symmetric in the *XY*, like a σ*-type orbital, that is strongly localized on the 1,1-dicyanomethylene ligand (Fig. 3b, up). This apparent inversion of symmetry will be fully discussed in the nitrogen K-edge. The second arises from an initial state of the two carbon atoms bonded with each other at the indanone, leading to π*-type final states that are predominantly distributed over the same unit (Fig. 3b, middle and down). On the higher-energy side, an additional peak labeled C′ exhibits similar characteristics, and it is visible in the TEY spectrum at high incidence angles, appearing around 286.7 eV. In the σ*-type transition, 1s electrons from the carbon in the C–S bonds are promoted to orbitals confined to the same unit, with a noticeable contribution from sulfur d-like states (Fig. 3c, up). In the π*-type transition, 1s electrons from the carbon atom adjacent to the C=O bond are promoted into the same indanone-localized final state observed for peak C (Fig. 3c, down). Both C and C′ consist of sets of transitions whose intensities evolve in opposite ways with angular variation. This behavior likely explains why neither feature exhibits a pronounced intensity change in the experimental spectra; in the case of C′, it is additionally masked by peak D.

In turn, peak D, similarly to peak E, is formed by a group of transitions with π*-type symmetry. One subset originates from excitation of 1s electrons from carbon in the C≡N groups into orbitals strongly localized on the ligand (Fig. 3d, middle-up), whereas the other arises from the carbon atoms in the C=C bonds of the thieno[3,2-*b*]thiophene unit into states that are slightly delocalized from this region toward the acceptor unit and/or the molecular core (Fig. 3d, middle-down). In the simulation, the separation of D and E is far more visible than in the actual experimental spectra. This leads us to think that the experimental energies of the transitions in the peak D are spread to higher energies, or that the experimental energies for peak E are spread to lower energies, either way, assisting the remarkable pronunciation of peak E in the grazing incidence measurements.

The broad peak F is the last feature within the π* region and is likewise composed of a series of transitions. In the theoretical M06 spectrum, two peaks were assigned to the experimental F feature, as they correspond to the highest-energy transitions that still exhibit π* symmetry. The lower-energy contribution (F1) arises from excitations of 1s electrons from the carbon in the C–S bonds into states that are slightly delocalized around the bond or extend toward the molecular core, as well as from the carbon atom of the indanone unit bonded to the 1,1-dicyanomethylene ligand into orbitals that are strongly localized either on this ligand or over the entire acceptor unit (Fig. 3f). The higher-energy contribution (F2) is formed by a single, well-defined transition, in which the carbonyl carbon is excited into a final state localized on the acceptor unit, with a density distribution very similar to that observed for the lower-energy component (Fig. 3g).

**Fig. 4:**
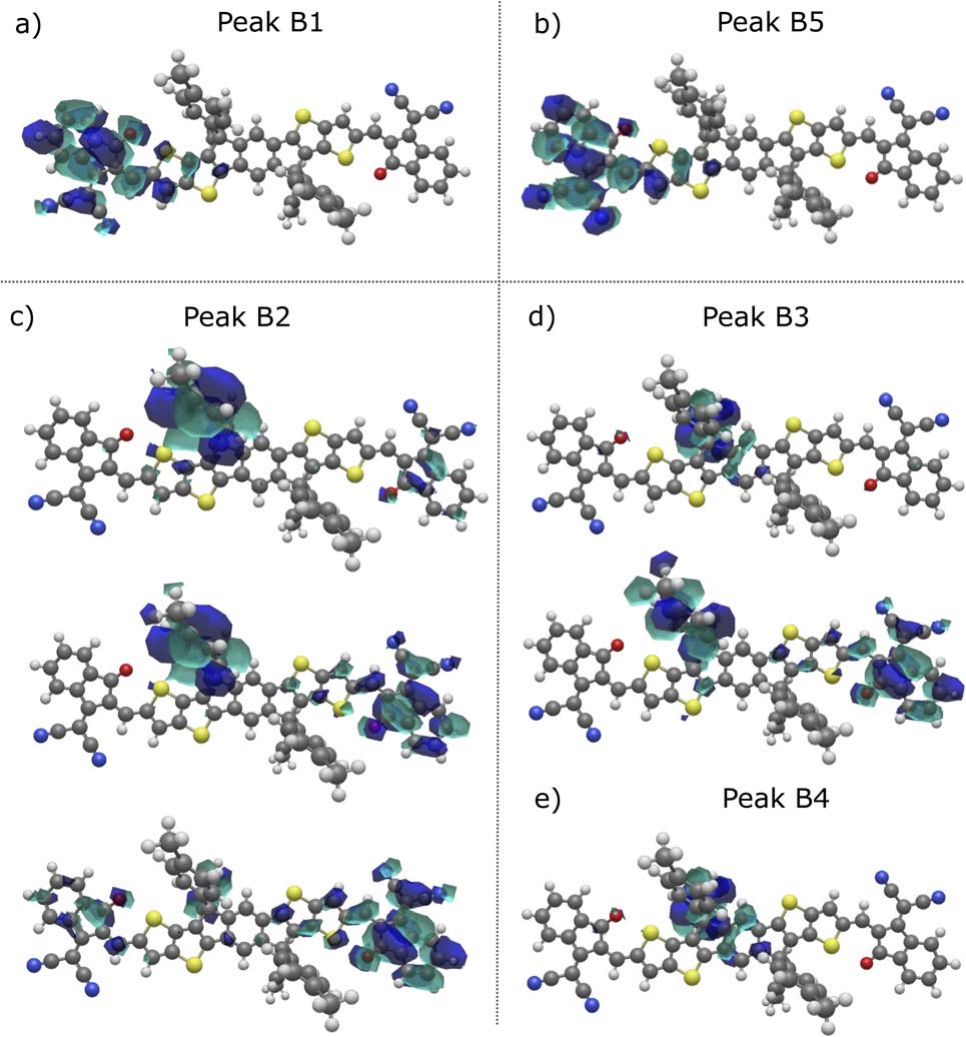
Final states natural transition orbitals (isovalue = 0.015) for the most representative transitions for peaks (a) B1, (b) B5, (c) B2, (d) B3, and (e) B4 in carbon K-edge NEXAFS spectra of ITIC simulated with M06/6-31G** level. Carbon atoms are colored in grey, hydrogen atoms in white, nitrogen atoms in blue, oxygen atoms in red, and sulfur atoms in yellow.

Peak B is perhaps one of the most intriguing assignments within this set of spectra. The small intensity oscillations clearly observed in the TEY spectrum could, at first glance, be attributed to experimental noise. However, the theoretical angular dependence results demonstrate that B is indeed composed of several distinct transitions (Fig. 2c), in which B1 and B5 increase toward grazing incidence, while B2, B3, and B4 decrease. In transitions B1 and B5, 1s electrons from the indanone carbon bonded to the 1,1-dicyanomethylene group and from the carbon in carbonyl, respectively, are excited to π* final states with very similar density distributions localized on the acceptor unit (Fig. 4a and b). In contrast, transitions B2, B3, and B4 originate from the carbons in the phenyl units in the side chains. Their corresponding final-state NTOs are primarily localized on the same phenyl rings and, in some cases, extend slightly into the core or acceptor unit (Fig. 4c–e).

Locally, the electron density on these phenyl units resembles antisymmetric π* orbitals within the ring plane. Due to the torsion angle in the molecular geometry, these phenyl rings are essentially perpendicular to the backbone and, consequently, perpendicular to the substrate plane (*XY*), causing the direction of these orbitals to lie parallel to the substrate, resulting in an intensity trend analogous to the σ* region. The angular effect is particularly pronounced in the theoretical spectra because the molecular backbone is perfectly aligned with the *XY* plane, and the bond angles of the side chains are all equal. In real samples, the measured signal represents an average over multiple molecular domains, and the chains possess some degree of freedom in torsion. Although the overall orientation should predominantly be parallel to the substrate (*D* < 0), local variations in tilt angles may enhance the overlap between the electric field and certain final states, partially restoring the “π* character” and preventing that kind of strong intensity of B2 at 90°. It is interesting to see that, in this case, the Gaussian convolution based solely on oscillator strengths (dashed line in Fig. 2c) produces an intensity pattern that makes it particularly difficult to rationalize the behavior of peak B in the experimental spectra. Incorporation of the angular dependence simulation was therefore essential to capture the observed nuances, significantly enriching the analysis.

#### 3.1.2 Oxygen

It is not unusual for oxygen K-edge spectra to be often challenging to interpret. One of the main issues is that oxygen is inherently associated with atmospheric contamination and is highly reactive with a wide range of surfaces, often leading to oxidation. Because NEXAFS measurements are performed under ultra-high vacuum, samples must be introduced into a load-lock chamber, where they are pumped down until the pressure equilibrates with the extremely low pressure of the analysis chamber (∼10^−9^ mbar). This procedure promotes the desorption of weakly adsorbed oxygen and other gaseous species from the surface, preventing their direct detection in the measurements.^12,15^ Nevertheless, chemical modifications induced by prior oxidation may still be reflected in the surface spectra. A second limitation when analyzing oxygen K-edge spectra for this type of sample is that many substrates commonly used for organic thin film depositions, such as ITO,^5–8,33,34^ FTO,^17^ and glass,^11,16^ contain oxygen in their composition, which may contribute to the measured signal if: (i) the film is too thin, (ii) the material isn't well distributed in the film and doesn't fully cover the substrate, (iii) or if the probing depth of the acquisition mode is too large.

In this work, the thickness of the ITIC and IDTBR films was measured by profilometry, resulting in (46 ± 9) nm and (31 ± 7) nm, respectively. Because they are sufficiently thin, the fluorescence spectra of both samples were compromised by strong oxygen signals from the substrate, as shown in Fig. S3.

Therefore, further discussion of these spectra will not be added in this work. However, survey spectra from X-ray photoelectron spectroscopy did not show signals from indium or tin in ITIC or IDTBR films (Fig. S4a and b), indicating that techniques sensitive only to the surface, such as NEXAFS measured in TEY mode, will only probe the organic thin film in both samples.

**Fig. 5:**
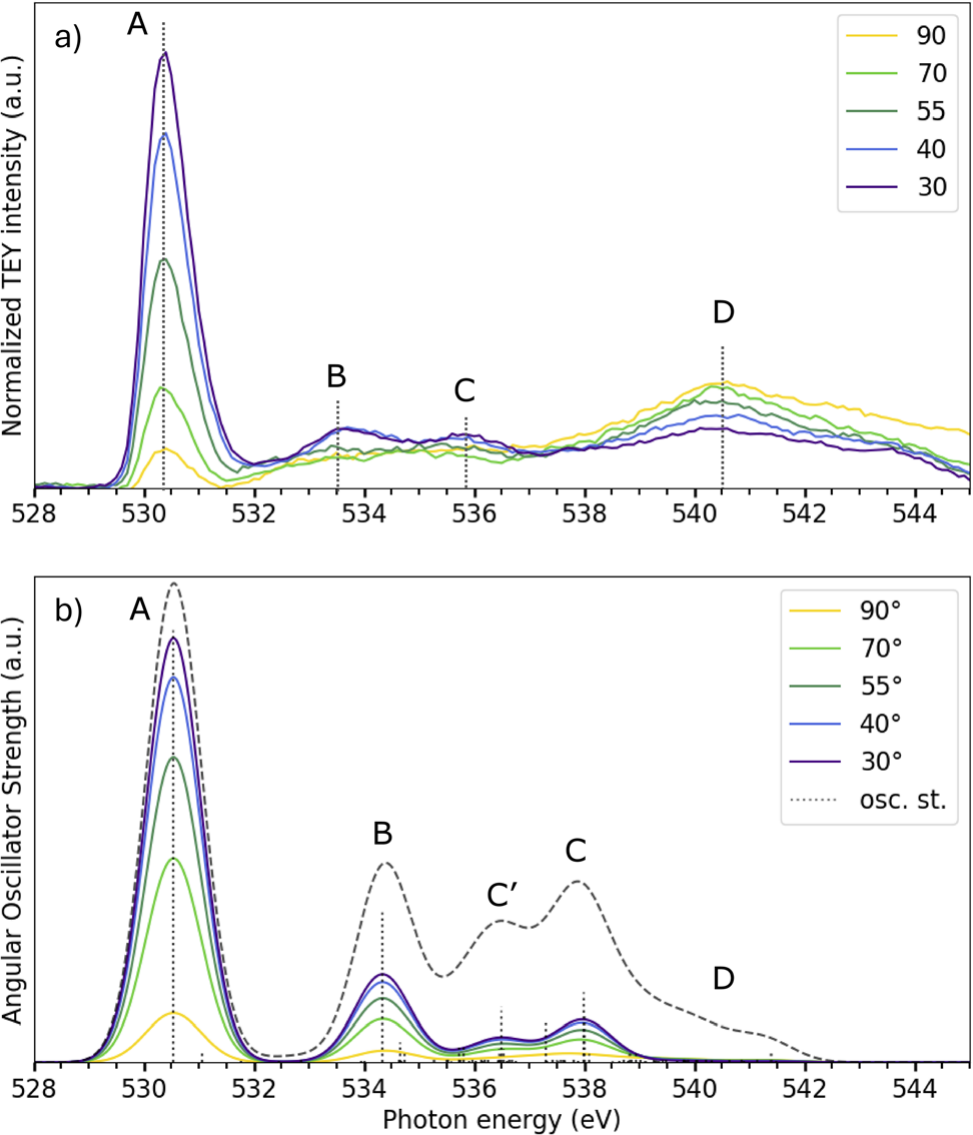
Experimental oxygen K-edge NEXAFS in (a) TEY, and (b) calculated oxygen K-edge NEXAFS obtained from TD-DFT calculation at M06/6-311G** theory level for ITIC.

Then, moving on with our discussion, a strong dichroism was observed in the oxygen K-edge TEY spectrum of ITIC (Fig. 5a). The π* region exhibits three main features at 530.3 eV (A), 533.5 eV (B), and 536.0 eV (C), all reaching the maximum intensity at the lowest incidence angle, whereas the σ* broadband centered around 540.5 eV (*D*) reaches its minimum. The resulting dichroic ratio for peak A is therefore negative, *D* = −0.83, strongly corroborating the face-on orientation suggested from the carbon spectrum.

**Fig. 6:**
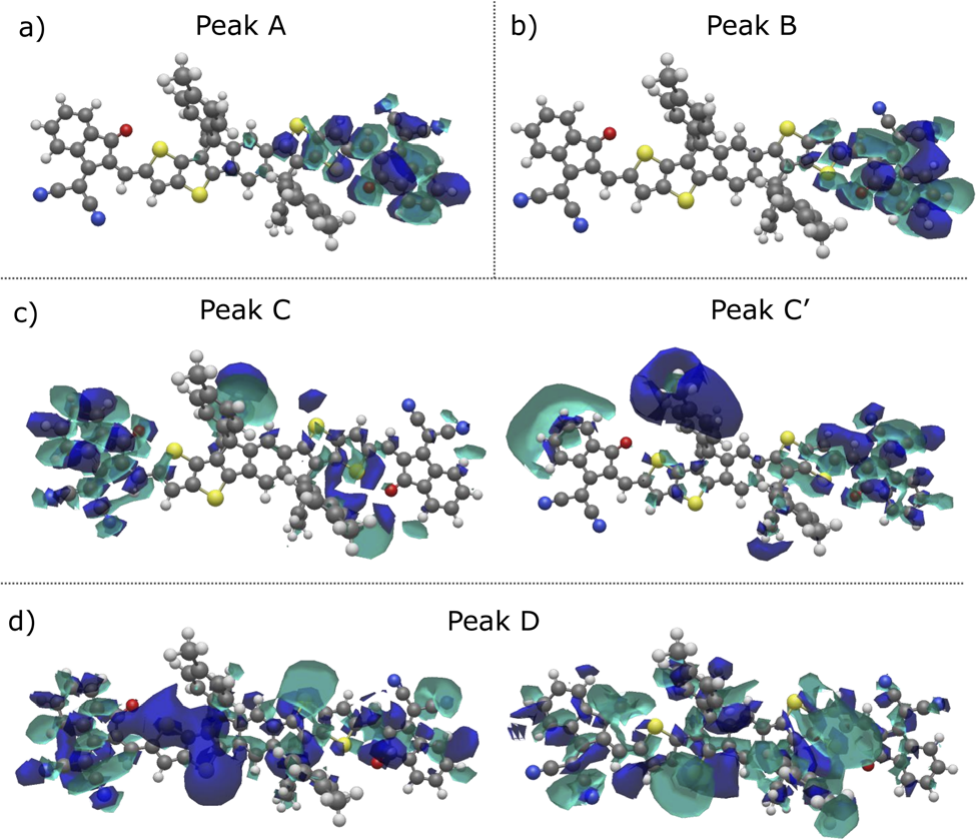
Final states natural transition orbitals (isovalue = 0.010) for the most representative transitions for peaks (a) A, (b) B, (c) C, and (d) D in oxygen K-edge NEXAFS spectra of ITIC simulated with M06/6-31G** level. Carbon atoms are colored in grey, hydrogen atoms in white, nitrogen atoms in blue, oxygen atoms in red, and sulfur atoms in yellow.

The theoretical spectra for all three functionals were consistent with the experimental results (Fig. 5b, S5a and b). Since the structure only has oxygen in a carbonyl bond, all calculated initial states originate from there. The final state of the first transition is the same unoccupied orbital observed for peak B5 in the carbon spectrum (Fig. 6a), which is strongly localized on the acceptor unit and partially on the thiophene linking it to the core. The final state associated with peak B appears here for the first time and is preferentially distributed over the indanone unit (Fig. 6b).

The experimental peak C is located between 535.0 and 537.0 eV and corresponds to the last feature of the π* manifold. The simulations indicate that this region is composed of two main contributions, labeled C and C′, spanning the 535.5–539.0 eV range. The final states associated with these transitions are more delocalized than those of peaks A and B (Fig. 6c). The NTOs show that the electron density is distributed over specific molecular fragments, with at least one acceptor unit exhibiting strong antisymmetry with respect to the *XY* plane, conferring π*-type character to these states. Peak D is formed by several final states that are delocalized over the entire molecule without a clear symmetry with respect to the *XZ* or *YZ* planes (Fig. 6d). Although these states are not perfectly symmetric with respect to the *XY* plane, they follow the trend of σ*-type orbitals upon simulation of the angular variation, in agreement with the experimental observations.

#### 3.1.3 Nitrogen

**Fig. 7:**
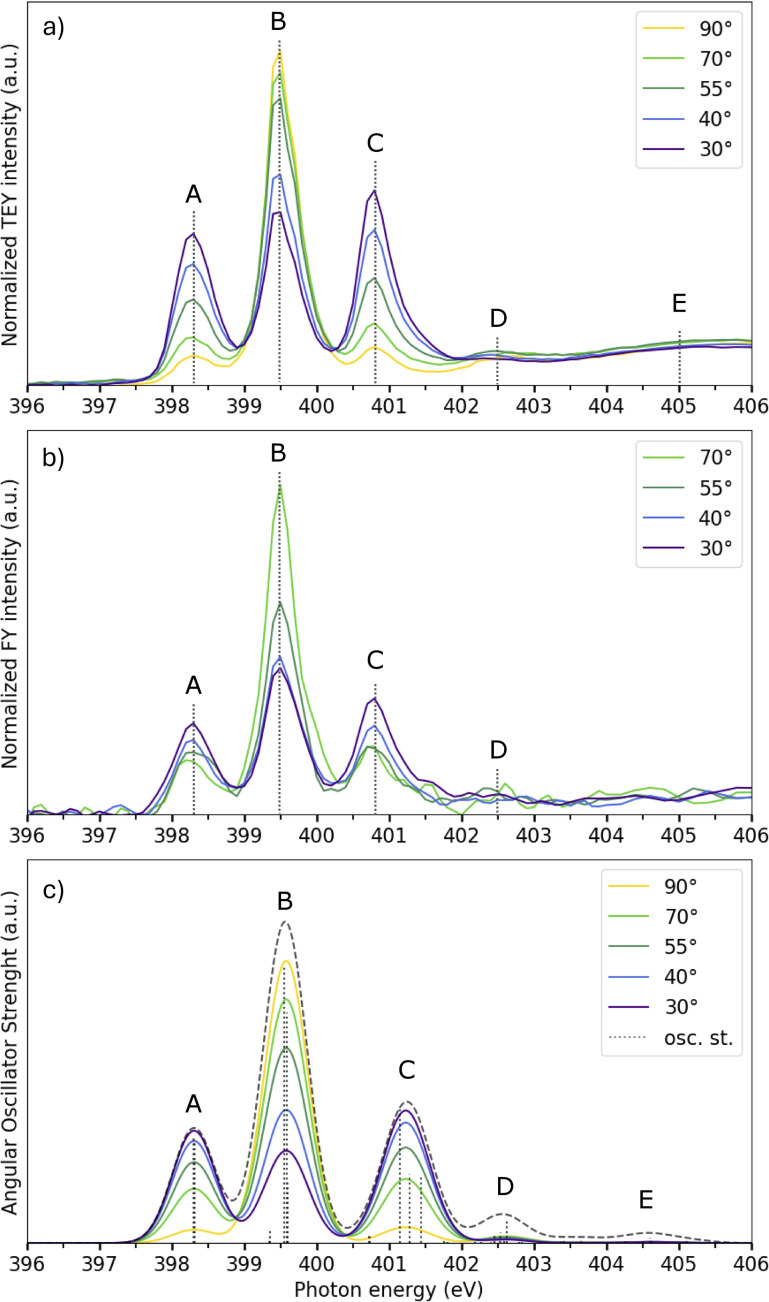
Experimental nitrogen K-edge NEXAFS in (a) TEY and (b) FY modes, and (c) calculated nitrogen K-edge NEXAFS obtained from TD-DFT calculation at M06/6-311G** theory level for ITIC.

The nitrogen K-edge spectrum (Fig. 7a and b) is characterized by three intense peaks at approximately 398.3 eV (A), 399.5 eV (B), and 400.8 eV (C), along with two weaker features around 402.5 eV (D) and 405.0 eV (E). The first transition was used to calculate the dichroic ratio, indicating a face-on orientation, as already inferred from the other edges, with *D* = −0.68 for the TEY mode and *D* = −0.28 for the FY mode.

An apparent inversion of the typical symmetry of the final states is also clearly observed at this edge. While the intensities of peaks A and C increase as the incidence angle decreases, peak B shows the opposite trend, which would be consistent with a σ*-type assignment. This type of phenomenon can be seen in edges whose probed atom is, for example, in a triple bond, since its nature is formed by one *σ* bond and two orthogonal π bonds, where one of them is in the same plane as the *σ*. These bonds generate antibonding orbitals in the same symmetry, resulting in at least one π* final state with low energy that appears in the NEXAFS spectrum with the same angular trend as an actual σ* resonance. This happens for other organic semiconductors, like Y5 and Y6 (a fluorinated derivative of Y5), since both molecules present the cyano group in their structure. Christopholi *et al.*^16^ show how their orientation in thin films changes when they are spin-coated with different organic solvents, and for that, the nitrogen K-edge was experimentally and theoretically investigated, exhibiting the same type of findings that we see here in ITIC.

**Fig. 8:**
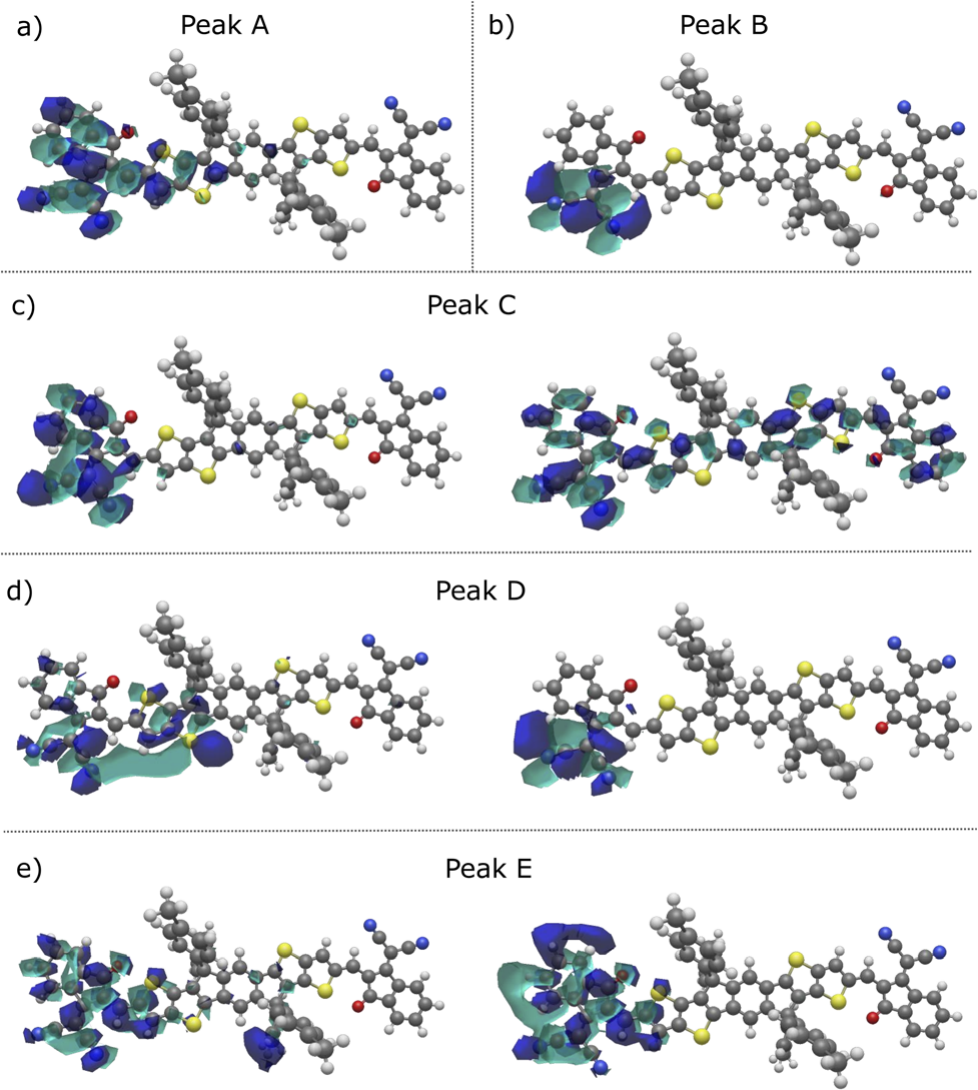
Final states natural transition orbitals (isovalue = 0.015) for the most representative transitions for peaks (a) A, (b) B, (c) C, (d) D, and (e) E in nitrogen K-edge NEXAFS spectra of ITIC simulated with M06/6-31G** level. Carbon atoms are colored in grey, hydrogen atoms in white, nitrogen atoms in blue, oxygen atoms in red, and sulfur atoms in yellow.

The overall shape of this spectrum is very well reproduced with the M06 functional (Fig. 7c). The final state associated with peak A closely resembles that of peak B1 in the carbon spectrum (Fig. 8a), being mainly composed of the LUMO and LUMO + 1. In contrast, the final state of peak B corresponds to a π* orbital, which has the symmetry plane as σ*-type states, localized around the C≡N bonds involving contributions from the LUMO + 11 and LUMO + 12. Therefore, these A and B final states exhibit antisymmetric and symmetric character with respect to the *XY* plane, respectively, corroborating the opposite intensity trends observed experimentally here, as well as for Y5 and Y6 in Christopholis' work.^16^

Peak C slightly deviates from the other features in this region, as it is formed by a set of transitions that are more widely spaced in energy. All its final states display π*-type symmetry and partially involve higher energy unoccupied orbitals, such as the LUMO + 28 and LUMO + 29 (Fig. 8c). Peak D exhibits final states with a clearly defined σ*-type symmetry, whereas peak E appears to arise from a mixture of symmetric and antisymmetric electron density distributions with respect to the *XY* plane (Fig. 8d and e). Both features display low intensity in the experimental spectra, particularly in the FY mode, and cannot be clearly distinguished.

Although all peaks involve final states whose electron density is predominantly localized on the acceptor unit, their relative intensities provide a clear illustration of the importance of the overlap between initial and final states in the understanding of the peak intensity. The more localized the orbitals on the 1,1-dicyanomethylene moiety are, the more intense the electronic transition. This also explains why peak C is slightly more intense than peak A. While the NTO associated with peak A spreads partially over the thiophene units of the donor moiety, the NTO of peak C, which is most favored by the angular variation, is more strongly localized on the donor unit. Even peak B, despite its σ*-type assignment, retains a significant intensity even at incidence angles that are unfavorable to this symmetry since its electronic density is only localized around the cyano groups.

### 3.2 IDTBR

#### 3.2.1 Carbon

**Fig. 9:**
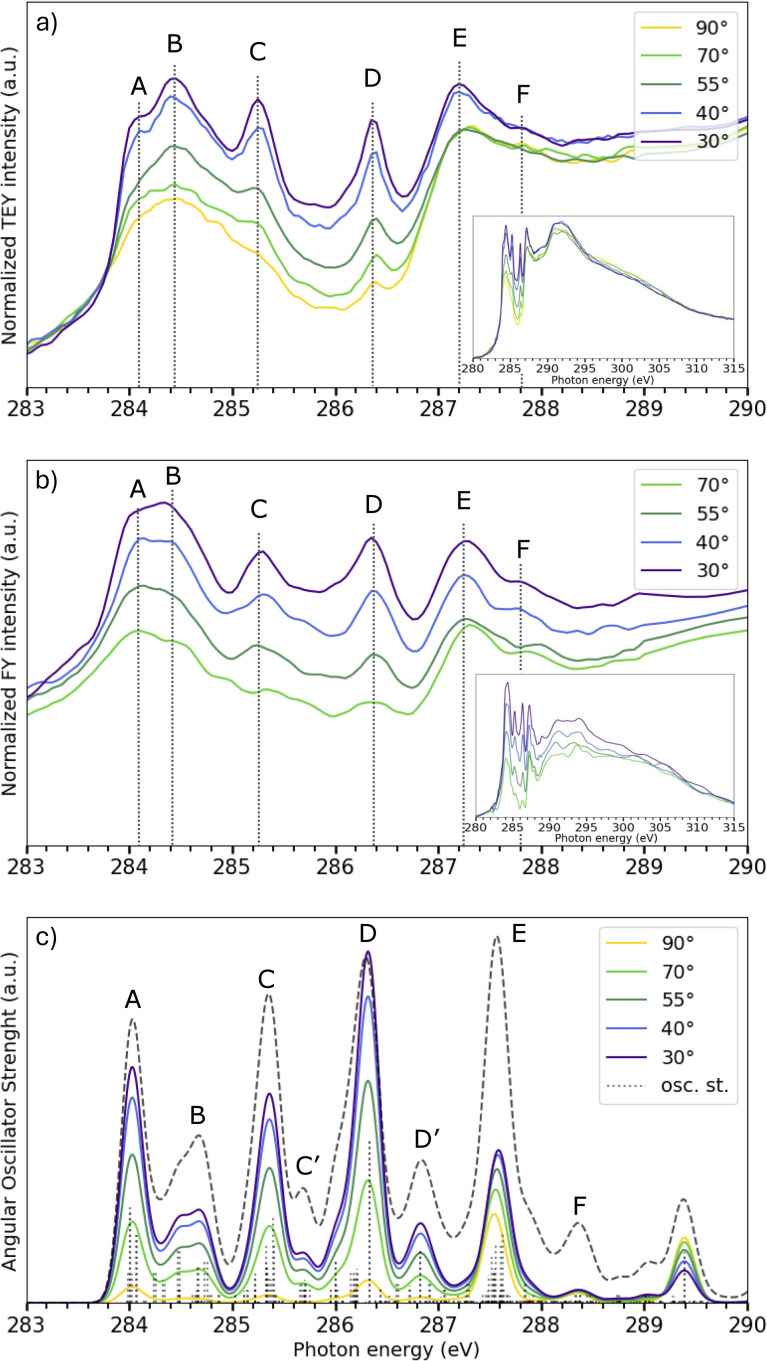
Experimental carbon K-edge NEXAFS in (a) TEY and (b) FY modes, and (c) calculated carbon K-edge NEXAFS obtained from TD-DFT calculation at M06/6-311G** theory level for IDTBR.

**Fig. 10:**
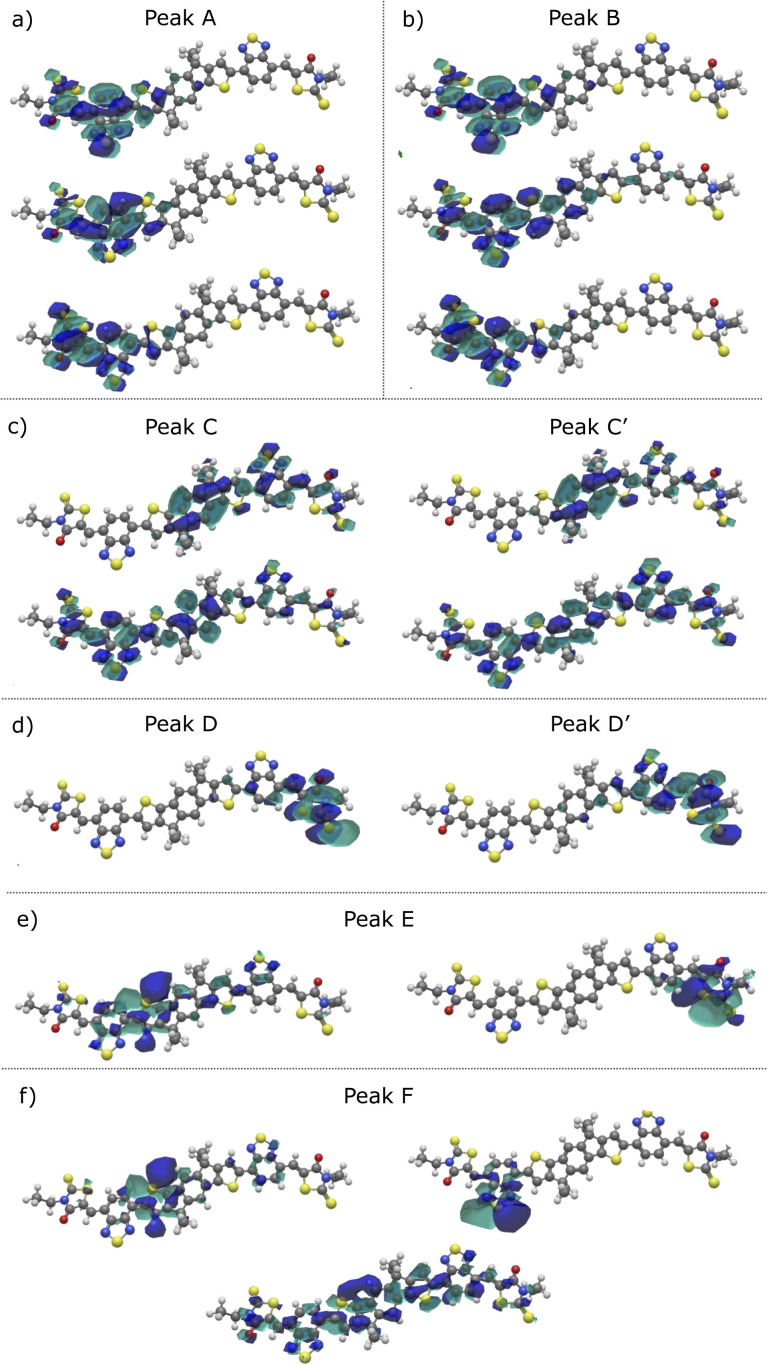
Final states natural transition orbitals (isovalue = 0.015) for the most representative transitions for peaks (a) A, (b) B, (c) C/C′, (d) D/D′, (e) E, and (f) F in carbon K-edge NEXAFS spectra of IDTBR simulated with M06/6-31G** level. Carbon atoms are colored in grey, hydrogen atoms in white, nitrogen atoms in blue, oxygen atoms in red, and sulfur atoms in yellow.

Unlike ITIC, the experimental carbon K-edge spectra of IDTBR exhibit a clear increase in overall intensity as the incident beam approaches grazing angles (Fig. 9a and b). Six features are observed in the π* region at approximately 284.1 eV (A), 284.4 eV (B), 285.2 eV (C), 286.4 eV (D), 287.2 eV (E), and 287.8 eV (F). In particular, the relative intensities of peaks C and D increase significantly compared to the other features, which can indicate a face-on type dichroism, as observed for ITIC, with dichroic ratios of *D* = −0.43 in TEY mode, and *D* = −0.74 in FY mode, calculated from peak D. The same trend is reproduced in the simulated spectrum (Fig. 9c), confirming the intensity enhancement at grazing incidence. This behavior arises because all dominant transitions exhibit an exclusive asymmetry with respect to the conjugated backbone (*XY* plane), characteristic of π*-type symmetry. Peaks A and B originate from sets of excitations of 1s electrons from the carbons in C=C bonds in the acceptor unit (rhodanine) and the extended acceptor unit (benzothiadiazole). As a consequence, the excited electron populates an orbital predominantly localized around these two regions. Their electron density distributions resemble mainly those of the LUMO + 2 and LUMO + 3 orbitals (Fig. 10a and b). Peak C arises from excitations of 1s electrons from carbon in C=C bonds in the core, populating final states delocalized between the core and one of the rhodanine units (Fig. 10c). Peak D is assigned to an excitation of 1s electrons from the carbon atom in the S–C=S groups, to a final state strongly localized on the rhodanine unit (Fig. 10d).

All final states contributing to peaks E and F resemble the non-occupied d orbitals from sulfur plus different contributions from neighboring atoms (Fig. 10e and f). In peak E, 1s electrons from carbon in C–S bonds in both the thiophene and rhodanine units are excited to orbitals localized around their respective moieties. In peak F, part of the transitions originates from excitations of 1s electrons from carbon in C=C bonds at the benzene of the benzothiadiazole unit, leading to NTOs delocalized over the core and with pronounced density on the thiophene sulfur. Another contribution arises from the excitations of 1s electrons from carbon in the C=N bond in the benzothiadiazole unit, yielding a state localized in the N–S–N linkage region. In FY mode, this transition is slightly better resolved and marginally more intense at higher incidence angles, reflecting the predominantly *XY*-symmetric character of the corresponding final-state NTO.

Peaks C′ and D′ cannot be unambiguously assigned in the experimental spectra. Most likely, their low intensities cause them to be masked by neighboring bands. Peak C′ originates from excitations of 1s electrons of carbon in the core region into highly delocalized final states, which explains its weak intensity due to poor orbital overlap (Fig. 10c). Peak D′, in turn, arises from excitations of 1s electrons in the carbonyl carbon to final states localized in the two moieties in the acceptor regions (Fig. 10d), which also contribute to one of the dominant final states associated with the experimental peak B.

#### 3.2.2 Oxygen

**Fig. 11:**
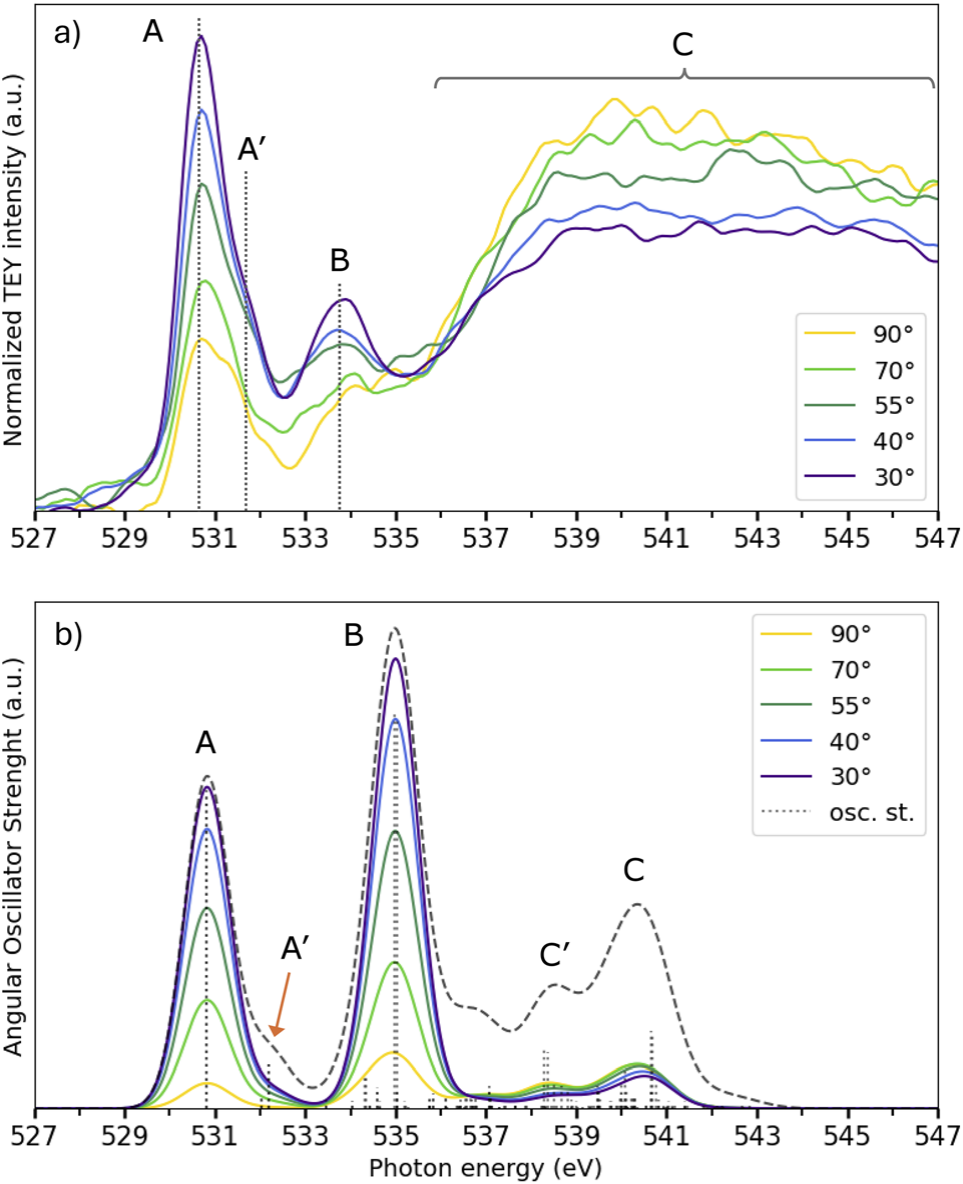
(a) Experimental oxygen K-edge NEXAFS in TEY mode, and (b) calculated oxygen K-edge NEXAFS obtained from TD-DFT calculation at M06/6-311G** theory level for IDTBR.

The π* region of the oxygen K-edge spectrum is composed of two main bands centered at approximately 530.8 eV (A) and 533.8 eV (B), along with a shoulder on the high-energy side of the main peak at 531.7 eV (A′) (Fig. 11a). The σ* region is identified as a single broad feature spanning the 536.0–547.0 eV range (C) (Fig. 11a). A strong dichroism is observed between the π* and σ* regions, with a dichroic ratio of *D* = – 0.47 calculated for peak A, corroborating a preferential face-on orientation of the molecule.

**Fig. 12:**
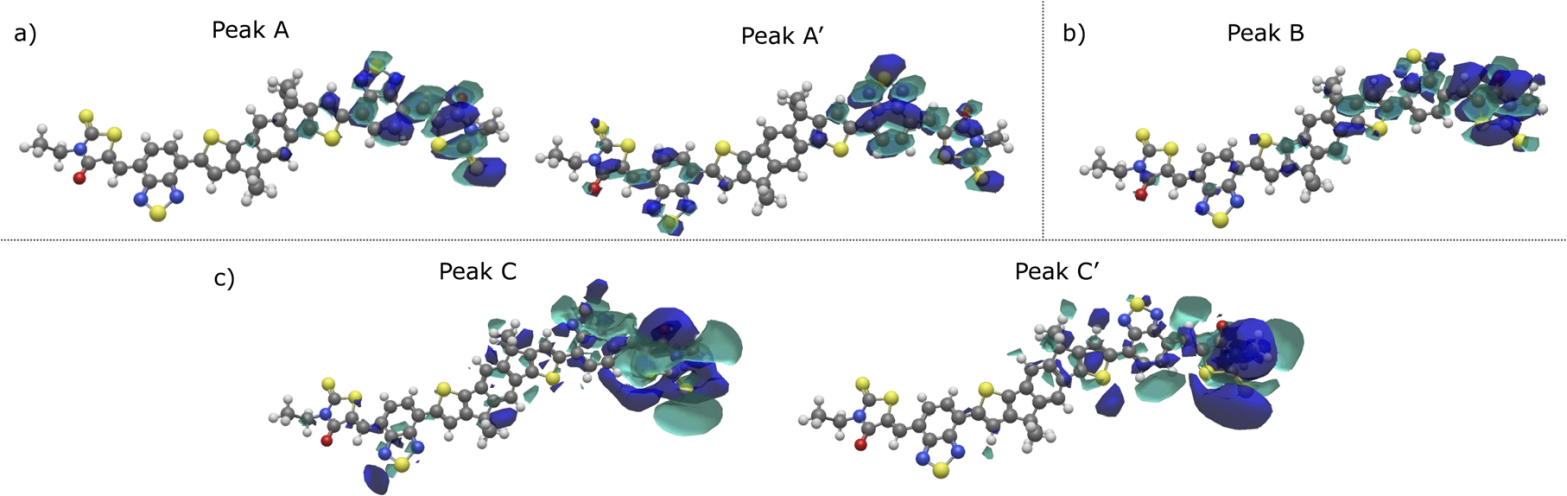
Final states natural transition orbitals (isovalue = 0.010) for the most representative transitions for peaks (a) A/A′, (b) B, and (c) C/C′ in oxygen K-edge NEXAFS spectra of IDTBR simulated with M06/6-31G** level. Carbon atoms are colored in grey, hydrogen atoms in white, nitrogen atoms in blue, oxygen atoms in red, and sulfur atoms in yellow.

In the simulated spectra, all experimental resonances are reproduced, and region C can be further resolved into two contributions, labeled C and C′ (Fig. 11b). The final-state NTOs are predominantly localized on the two acceptor units. As in ITIC, the oxygen atom in the IDTBR only appears in a C=O bond, so all initial states come from the same type of oxygen. The final state from peak A is the same NTO associated with peak D′ in the carbon K-edge spectrum (Fig. 12a). The final state corresponding to A′ resembles those observed for transitions A and B in the carbon spectrum and shows pronounced symmetry with respect to the *ZY* plane of the benzothiadiazole unit (Fig. 12a). For peak B, the final state density is strongly concentrated on the rhodanine, with partial delocalization extending into the core (Fig. 12b). In the states contributing to region C, σ*-like symmetry is observed, originating mainly from the rhodanine moiety (Fig. 12c).

#### 3.2.3 Nitrogen

**Fig. 13:**
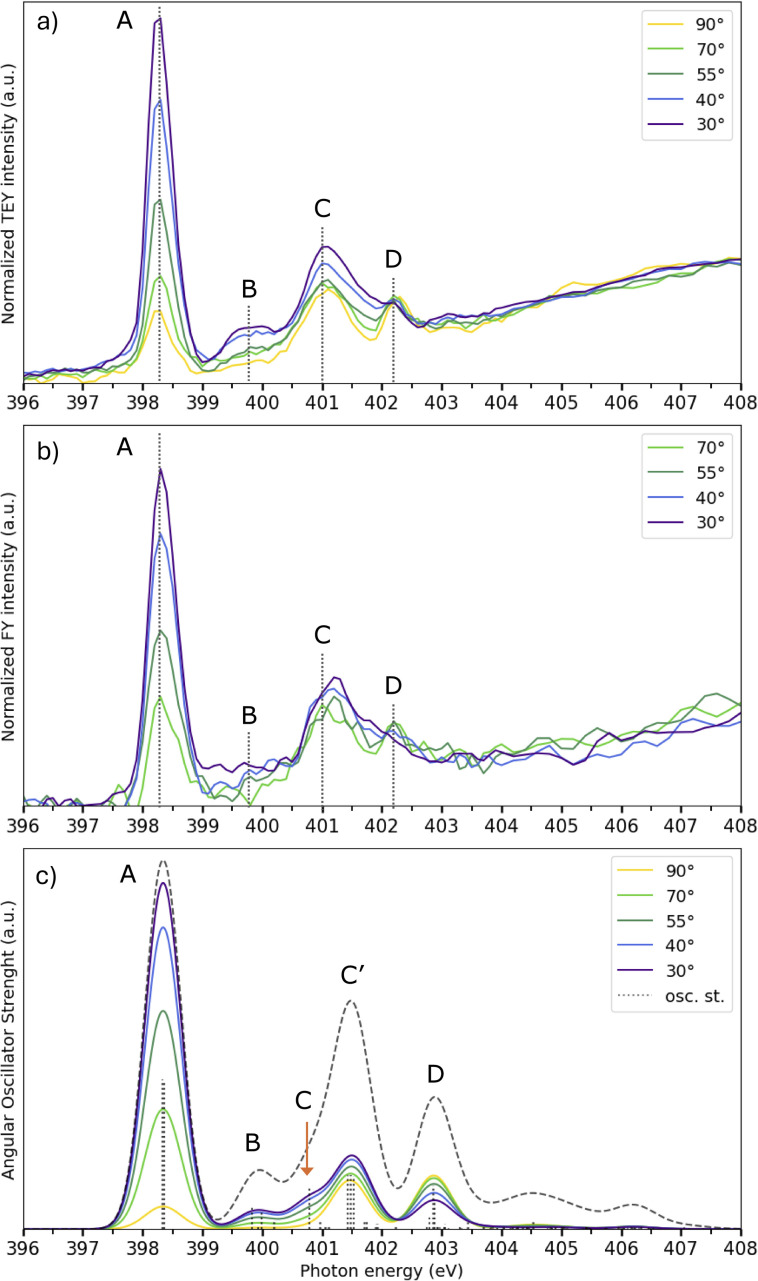
Experimental nitrogen K-edge NEXAFS in (a) TEY and (b) FY modes, and (c) calculated nitrogen K-edge NEXAFS obtained from TD-DFT calculation at M06/6-311G** theory level for IDTBR.

**Fig. 14:**
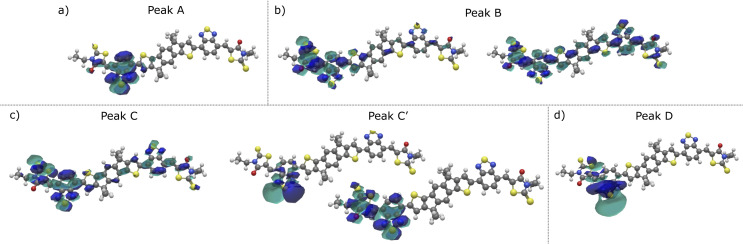
Final states natural transition orbitals (isovalue = 0.010) for the most representative transitions for peaks (a) A/A′, (b) B, and (c) C/C′ in nitrogen K-edge NEXAFS spectra of IDTBR simulated with M06/6-31G** level. Carbon atoms are colored in grey, hydrogen atoms in white, nitrogen atoms in blue, oxygen atoms in red, and sulfur atoms in yellow.

Fig. 13 shows that four main features can be identified in the nitrogen K-edge spectra of IDTBR. The peaks at 398.3 eV (A), 399.7 eV (B), and 402.2 eV (D) originate exclusively from transitions involving nitrogen atoms in the benzothiadiazole. The final-state associated with peak A is antisymmetric with respect to the molecular *XY* plane (π*-type) and highly localized on the benzothiadiazole moiety (Fig. 14a). As a result, the transition intensity increases at grazing incidence, yielding a dichroic ratio of *D* = −0.67 for TEY mode, and *D* = −0.51 for FY mode, which supports the face-on dichroism inferred from the carbon and oxygen K-edge spectra.

The spectral region corresponding to peak B is composed of two dominant transitions to π*-type final states with higher electron density on the two acceptor units while remaining delocalized over the molecule (Fig. 14b). This behavior is similar to that observed for peak C′ in the carbon spectrum and explains the comparatively low overall intensity of this band. In contrast, for peak D, the electron density is strongly concentrated on the N–S–N linkage and is symmetric with respect to the *XY* plane, giving rise to a σ*-type character (Fig. 14d). Consequently, the intensity of this transition decreases as the incidence angle is reduced. This effect is only subtly observed in both experimental spectra because the asymmetric growth of band C at 401.0 eV toward higher energies nearly obscures peak D at an incidence angle of 30°. This overlap is not reproduced in any of the theoretical spectra (Fig. 13c, S9a and b), as peak D is shifted towards higher energy in the simulations.

Peak C is the only feature that arises from a mixture of transitions originating from both nitrogen chemical environments. Two sets of transitions in the M06 theoretical spectrum (Fig. 13c) correspond to the third experimental peak C: one centered around 401.0 eV (C), and the other around 401.5 eV (C′). In C, the initial state originates from nitrogen atoms in the rhodanine unit, and the final state exhibits symmetry and electron density distributions similar to the average of those associated with peak B (Fig. 14c). Peak C′, however, comprises two distinct transitions that occur at nearly the same energy. In one case, 1s electrons from the rhodanine nitrogen are excited to a π*-type region strongly localized on the two acceptor units (Fig. 14c). In the other, 1s electrons from the benzothiadiazole nitrogen are excited to the same final-state NTO associated with peak F in the carbon spectrum, which has σ*-type symmetry (Fig. 14c). Therefore, the increase in intensity observed for this band is mainly driven by the first set of transitions.

## 4. General discussion

Before diving into a detailed discussion of dichroic ratios and how the simulated angular variation spectra can contribute to this analysis, it is necessary to justify the use of a hybrid *meta*-GGA functional like M06. Calculations for all edges in both materials were also performed using the hybrid functional B3LYP and the GGA functional PBE, which are widely employed in the literature.^19–24,26^

The decrease in accuracy to reproduce the carbon K-edge with increasing spectral complexity becomes evident when moving from M06 to B3LYP, and even more markedly to PBE. For ITIC, B3LYP provides a less satisfactory description of peak B, with excessive separation of B1 and an inadequate reproduction of angular intensities in B3–B4 (Fig. S2a), while PBE shows even larger discrepancies, making the peak assignment less reliable as it overestimates energies, and leads to incorrect angular ordering (Fig. S2b). For IDTBR, the superiority of M06 over B3LYP becomes clearer, allowing a more straightforward assignment of experimental resonances, whereas B3LYP exhibits excessive overlap (Fig. S6a), which is enhanced when broader spectral widths are used in the simulations (Fig. S7). Both hybrid functionals, however, overestimate energies and underestimate the intensities of peak B, with a slight energy shift in M06. PBE again fails to reproduce the spectrum, suppressing low-energy peaks and generating inconsistent intensity for the features in the spectrum (Fig. 6b). This failure is consistent with such type of functional, since the lack of exact exchange correction in the model leads to great self-interaction error in localized core excitations.^37^

For the oxygen K-edge, all functionals show a similar systematic limitation in overestimation of the energy separation between peaks A and B, resulting in an overall shift of the remaining spectra; besides that, they provide a reasonable spectral description. More specific observations about their accuracy are given as follows. For ITIC, B3LYP (Fig. S5a) better reproduces relative intensities within the B–C′–C region, while PBE (Fig. S5b) more accurately captures peak spacing due to its tendency to underestimate transition energies. Additionally, it enhanced the intensity of peak D. For IDTBR, a similar overestimation of the A–B separation is observed, although the general spectral shape and angular dependence are well reproduced by all. In hybrid functionals (Fig. 11b and S8a), peak B is predominantly described by a single intense transition. Experimentally, however, this peak exhibits noticeable broadening toward lower energies, which may originate from weak transitions between 534.0 and 535.0 eV observed in the theoretical spectra; this contribution is better captured by B3LYP than by M06. In contrast, PBE assigns significant weight to these lower-energy transitions but fails to reproduce the A′ shoulder with the expected intensity (Fig. S8b).

For the nitrogen K-edge, B3LYP (Fig. S9a and S10a) and M06 produced nearly identical results for both IDTBR and ITIC, accurately reproducing experimental energies and relative intensities. In contrast, PBE shows noticeable deviations: for ITIC (Fig. S10b), peaks B and C are shifted to higher energies and partially merge due to near-degeneracy with peak D, while intensity trends are also misrepresented, demonstrating how small energy errors can significantly distort the overall spectral shape. For IDTBR (Fig. S9b), the main limitation of PBE is the appearance of an additional low-energy feature (A′) associated with delocalized excitations, which may lead to misassignment of the first peak. Furthermore, the shift toward high energy of peak A reduces the resolution of the subsequent feature. Despite these issues, the application of the simulation of angular dependence improves the qualitative agreement of PBE, particularly in reproducing relative intensity trends more consistently with hybrid functionals. Overall, M06 outperforms the other functionals and has to be used in this work for the comparative analysis.

In respect of molecular orientation, the experimental angular dependence of the NEXAFS spectra in TEY and FY modes for both molecules consistently indicates a preference for a face-on orientation. To quantify this dichroism, the dichroic ratio (*D*) can be calculated, usually using the intensity of the first π* transition of the spectrum. Even though all transitions share the same symmetry, the spatial distribution of the unoccupied states, which will be populated by the excited core electron, can lead to different *D* values, even when all probed edges belong to atoms along the same molecular backbone and would therefore be expected to experience the same degree of orientation.

For the TEY spectra of ITIC, the experimental *D* values for peak A at the carbon, nitrogen, and oxygen K-edges are −0.44, −0.68, and −0.83, respectively, while the theoretical *D* values obtained from simulated spectra with an artificial electric field are −0.74, −0.75, and −0.80. From a mathematical point of view, the oscillator strength does not affect the dichroic ratio expression, as it cancels out. Therefore, *D* depends exclusively on the interaction between the beam electrical field and the cartesian components of the dipole moments, *L*_*x*_, *L*_*y*_, and *L*_*z*_, of the transitions that are more relevant to the peak. The modulus of these components, |*L*_*x*_|, |*L*_*y*_|, and |*L*_*z*_|, as well as the magnitude of the dipole vector, *L*^2^, for the most intense transitions associated with the theoretical peaks used to calculate *D* in ITIC, are listed in Table 1. We observe that *L*^2^ is more than twice as large for carbon compared to the other edges, but this does not appear to be a relevant factor, since the theoretical *D* values for all edges are sufficiently similar. What makes them comparable is the ratio between the magnitudes of *L*_*x*_ and *L*_*y*_ relative to *L*_*z*_, which is, on average, about 8.5 times larger than the in-plane components, regardless of the edge analyzed. The theoretical *D* values are numerically consistent with the nitrogen and oxygen spectra, but not with carbon (dispenses the negative value). This incompatibility of the result with other edges requires further consideration and will not be discussed here.

**Table 1:** *L* ^2^, |*L*_*x*_|, |*L*_*y*_|, and |*L*_*z*_| in atomic units (a.u.) of the main transition dipole moments associated with the first π* transition (most intense oscillator strength in peak A) in the ITIC carbon, nitrogen, and oxygen K-edge spectra

ITIC K-edge	*L* ^2^ (a.u.)	|*L*_*x*_| (a.u.)	|*L*_*y*_| (a.u.)	|*L*_*z*_| (a.u.)
Carbon	0.00329	0.00676	0.00725	0.05648
	0.00329	0.00685	0.00611	0.05665
Nitrogen	0.00139	0.00427	0.00497	0.03673
	0.00139	0.00355	0.00399	0.03696
	0.00139	0.00380	0.00391	0.03642
	0.00136	0.00435	0.00497	0.03625
Oxygen	0.00134	0.00374	0.00406	0.03613
	0.00135	0.00395	0.00402	0.03623

In fluorescence yield mode, the dichroic ratios for carbon and nitrogen in ITIC are −0.32 and −0.28, respectively. This clear decrease in *D* for both edges indicates that the degree of face-on packing in the bulk is lower than at the surface. Since no positive *D* values are observed, it is more likely that molecules in the bulk are simply more disordered rather than adopting a preferential edge-on orientation. Notably, the degree of orientation expressed by *D* between TEY and FY is relatively small for carbon, but much larger for nitrogen. These results highlight the importance of analyzing multiple edges and acquisition modes to reach a more accurate conclusion about sample morphology.

For IDTBR, calculating *D* from peak A in the carbon K-edge is impractical, particularly in the FY spectrum, as this transition significantly overlaps with peak B. Therefore, peak D was selected instead, as it is better defined in the spectrum. For TEY spectra, the experimental *D* values for carbon, nitrogen, and oxygen are −0.43, −0.67, and −0.47, respectively, whereas for fluorescence spectra, they are −0.74 and −0.51 for carbon and nitrogen. Here, differences are observed between surface and bulk: the carbon spectrum suggests that the bulk is more prone to a face-on orientation, while the nitrogen spectrum indicates that the bulk is somewhat less organized than the surface. Unlike ITIC, where all the final states of the selected peaks are localized in the acceptor unit, in this case, the electron density of the nitrogen peak A is highly concentrated in the extended acceptor unit (benzothiadiazole), while that of the carbon peak D is localized in the acceptor unit (rhodanine). This may indicate a difference in backbone torsion between surface and bulk molecules, which could influence the spectral response, although it does not directly reveal whether there is a significant difference in the degree of face-on orientation between these regions.

The theoretical *D* values calculated for carbon, oxygen, and nitrogen are −0.88, −0.85, and −0.89, respectively, significantly larger in magnitude than the experimental values. Analysis of the dipole components in Table 2 shows that most of the *L*_*y*_ values are significantly larger than in ITIC. Although *L*_*z*_ is still dominant (on average about five times larger), this increase in *L*_*y*_ enhances the contribution of the in-plane components, making the overlap with the artificial angular vector more relevant. As a result, both extreme directions – normal and grazing incidence – become more pronounced, increasing their difference and, consequently, the theoretical dichroic ratio. This may indicate that, although a face-on preference exists, IDTBR remains mostly disordered, particularly at the surface.

**Table 2:** *L* ^2^, |*L*_*x*_|, |*L*_*y*_|, and |*L*_*z*_| in atomic units (a.u.) of the main transition dipole moments associated with π* transitions in the IDTBR carbon (peak D), nitrogen (peak A), and oxygen (peak A) K-edge spectra

IDTBR K-edge	*L* ^2^ (a.u.)	|*L*_*x*_| (a.u.)	|*L*_*y*_| (a.u.)	|*L*_*z*_| (a.u.)
Carbon	0.00621	0.00599	0.03273	0.07146
	0.00622	0.00213	0.00983	0.07822
Nitrogen	0.00280	0.00361	0.01992	0.04894
	0.00280	0.00195	0.01007	0.05188
	0.00273	0.00193	0.00985	0.05128
	0.00274	0.00343	0.01959	0.04843
Oxygen	0.00085	0.00109	0.00405	0.02893
	0.00087	0.00254	0.01249	0.02659

## 5. Conclusions

In this work, we provided a detailed description of the C, N, and O K-edge NEXAFS spectra of the non-fullerene acceptors ITIC and IDTBR, combining with TDDFT calculations. In what concerns the theory level, hybrid functionals M06 and B3LYP consistently outperformed PBE. However, PBE showed reasonable accuracy for simpler absorption edges, *i.e.*, atoms with only one chemical environment, like all oxygen K-edges and the nitrogen K-edge of ITIC. Also, its qualitative description could be notably improved through the simulation of the angular dependence. For a detailed description of electronic transitions much closer in energy, like in the carbon spectrum, M06 performed better than B3LYP, making it a better choice for the theoretical description of the edges we have analyzed.

The outcomes of this study also demonstrate that angular-dependent NEXAFS is an indispensable tool, not only for determining preferential molecular orientation, but also for a more complete description of the electronic structure by assigning the nature of the unoccupied states. For this, we perform a data treatment in the calculated spectra that simulates the effect of the polarized electric field, providing insightful information and resulting in an excellent agreement with experimental spectra. In particular, for ITIC, where π* final states symmetric in the *XY* plane (resembling a σ* character) were identified, the angular dependence of the electronic transitions significantly facilitated spectral assignments in the carbon and nitrogen K-edges. This apparent “symmetry inversion” was observed primarily for final states whose electronic density is strongly concentrated around the sulfur atoms (in both ITIC and IDTBR) and on the cyano group of ITIC. In the case of sulfur, this σ*-like character arises largely from contributions of d-like atomic orbital for the density of unoccupied states. The angular dependence observed at the nitrogen K-edge of ITIC reflects the intrinsic nature of the C≡N triple bond, where transitions into orthogonal π*-type states contribute to the resonance. The variation of the X-ray incidence angle can also be crucial to enhance transitions with a stronger contribution from delocalized final states, which generally exhibit lower intensity. The most evident example is peak B (399.7 eV) in the nitrogen K-edge spectrum of IDTBR, where the transition is essentially absent at normal incidence but becomes clearly visible as the angle of incidence approaches the grazing configuration.

The experimental and theoretical dichroic ratios for different edges in the same molecule were compared. TEY and FY spectra in the ITIC results indicate a higher degree of face-on organization at the surface than in the bulk. In contrast, IDTBR presents a more complex scenario, where different trends were observed between surface- and bulk-sensitive measurements, depending on the edge probed. These differences can be attributed to the localization of the excited states of the chosen peak for the dichroic ratio calculations, implying different degrees of torsion in the molecular backbone between bulk and surface. Theoretical dichroic ratios for nitrogen and oxygen in ITIC spectra were comparable with the TEY measurements, indicating that on the surface, the tilt angle is probably close to the plane parallel to the substrate, since the theoretical spectra were simulated with the ITIC in a perfect parallel alignment with the *XY* plane. For IDTBR, the theoretical *D* values were higher than the experimental measurements, suggesting that, despite a tendency toward face-on orientation, IDTBR exhibits a higher degree of structural disorder than ITIC. Comparing between molecules, the theoretical *D* becomes higher when the in-plane angular components show a comparable magnitude to the *L*_*z*_. In ITIC, the average theoretical *D* is −0.76 for a *L*_*z*_ that is 8.5 times higher than *L*_*x*_ and *L*_*y*_, while in IDTBR, the average theoretical *D* is −0.87 for a *L*_*z*_ that is around 5 times bigger than *L*_*y*_, and around the same as ITIC for *L*_*x*_.

This study demonstrates that a combined angular-dependent NEXAFS analysis for different edges and different depth ranges, supported by a computational approach, enables a detailed and physically consistent interpretation of core-level excitations in conjugated organic molecules. By correlating spectral features with the symmetry and localization of unoccupied states across carbon, nitrogen, and oxygen K-edges, we demonstrate how complementary information from different edges can be used to unravel complex electronic responses. This approach provides a reliable framework for interpreting NEXAFS spectra of organic semiconductors and for assessing subtle changes in their electronic structure that are directly relevant to optoelectronic devices. As an outlook, we intend to apply this theoretical angular analysis to donor polymers and analyze the experimental angular-dependent NEXAFS spectra in blend films with different acceptors, discussing how this approach can help in peak assignments.

## Author contributions

Beatriz M. Guerra: conceptualization, methodology, software, formal analysis, investigation, data curation, visualization, writing – original draft. Marcin Zając: investigation, writing – review and editing. Andreas Opitz: investigation, writing – review and editing. Cleber F. N. Marchiori: conceptualization, investigation, data curation, writing – review and editing, supervision, funding acquisition. Maria Luiza M. Rocco: conceptualization, investigation, writing – review and editing, supervision, funding acquisition.

## Conflicts of interest

There are no conflicts to declare.

## Supplementary Material

RA-016-D6RA03796H-s001

## Data Availability

The raw spectroscopic data and computational input files supporting this study are available from the corresponding author upon reasonable request. Supplementary information (SI): optimized molecular geometries, simulated angular-dependent NEXAFS spectra obtained using the B3LYP and PBE functionals for the carbon, nitrogen, and oxygen K-edges of both molecules, oxygen K-edge NEXAFS spectra acquired in fluorescence yield mode, and XPS survey spectra. See DOI: https://doi.org/10.1039/d6ra03796h.
