# Supplementary material for: Multi-edge NEXAFS study of non-fullerene acceptors: electronic structure and molecular orientation supported by simulations of NEXAFS angular dependence
Source: RSC Adv. 2026 Jul 3;16(35):36650–64. doi: 10.1039/d6ra03796h (PMC13330984; doi:10.1039/d6ra03796h)
Supplement: RA-016-D6RA03796H-s001 [file RA-016-D6RA03796H-s001.pdf]

Supplementary Information (SI) for RSC Advances.
This journal is © The Royal Society of Chemistry 2026

SUPPORT INFORMATION

Multi-edge NEXAFS study of non-fullerene acceptors: electronic structure and molecular orientation supported by simulations of NEXAFS angular dependence

Beatriz Molinaro Guerra,[a] Marcin Zając,[b] Andreas Opitz,[c] Cleber F. N. Marchiori,*[d] Maria Luiza M. Rocco*[a]

Adress:

a. Institute of Chemistry, Federal University of Rio de Janeiro (UFRJ), Rio de Janeiro, RJ, Brazil.
b. National Synchrotron Radiation Centre SOLARIS, Jagiellonian University, Kraków, Poland
c. Institut für Physik, Humboldt-Universität zu Berlin, Berlin, Germany
d. Department of Engineering and Physics, Karlstad University, Karlstad, Sweden.

Corresponding author email: cleber.marchiori@kau.se, luiza@iq.ufrj.br

1 - Computational details

The structures of ITIC and IDTBR were constructed in Avogadro, and they were used as an input for the optimization in the three levels of theory: PBE/6-311G**, B3LYP/6-311G** and M06/6-311G**. Since M06 functional results in the better performance in TDDFT, below we choose to report only the optimized geometries of ITIC and IDTBR (Figure S1) obtained with this functional, and their respective molecular coordinates in. xyz format.

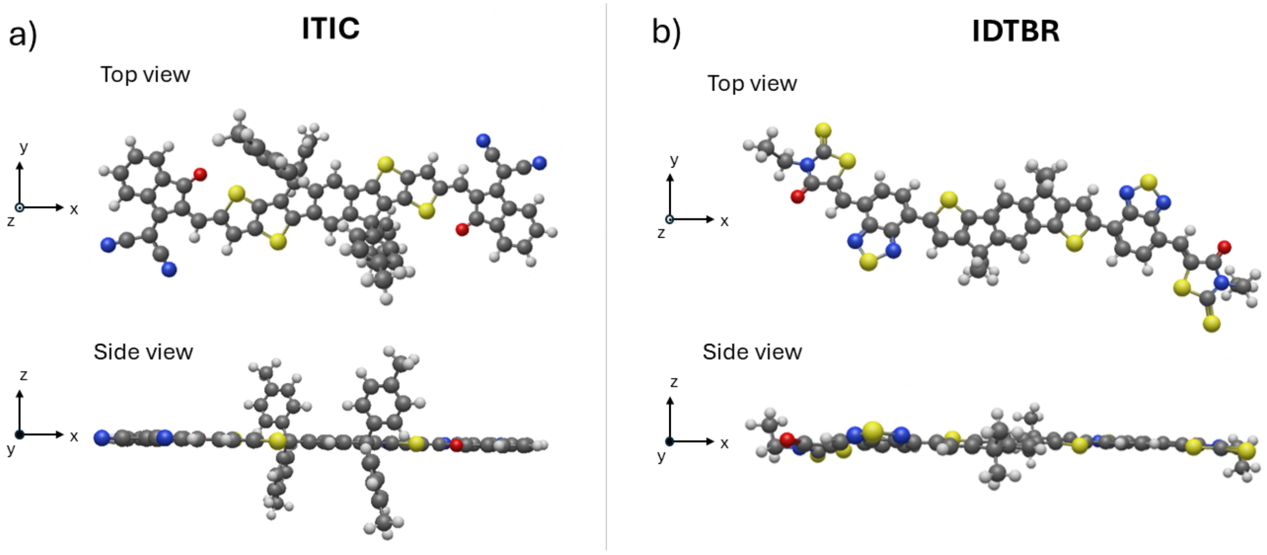

Figure S1. Optimized geometries for (a) ITIC and (b) IDTBR obtain by M06/6-311G** theory level.

Optimized Geometry of ITIC by M06/6-311G**

126

XYZ file generated by orca_plot on BaseName=ITIC

C	-5.422343	1.160015	-0.232445
C	-6.065785	-0.074808	-0.459586
C	-4.054365	1.219240	-0.072604
C	-3.348984	0.017660	-0.119148
H	-3.545615	2.164909	0.098200
C	-3.994855	-1.218717	-0.329184
C	-5.359524	-1.275529	-0.513610
H	-5.868632	-2.222117	-0.679063
C	-1.945314	-0.268143	0.030185
C	-1.704273	-1.616455	-0.083682
C	-2.995636	-2.376660	-0.310825
C	-3.188658	-3.317905	0.880033
C	-3.050050	-3.090366	-1.661001
C	-6.428625	2.312543	-0.207252
C	-7.714652	1.554958	-0.468233
C	-6.075829	3.291139	-1.326923
C	-6.549965	2.981511	1.163327
C	-7.471143	0.208466	-0.595596
S	-0.537726	0.698178	0.328795
C	0.431169	-0.762153	0.303078
C	-0.341050	-1.911672	0.079872
S	-8.879022	-0.759294	-0.887450
C	-9.850416	0.698902	-0.846864
C	-9.081455	1.846513	-0.603671
C	-6.619389	3.157801	-2.600054
C	-6.233103	4.003665	-3.632501
H	-7.355784	2.383215	-2.797489
C	-5.290922	5.005247	-3.429106
H	-6.677467	3.880567	-4.617138
C	-4.741995	5.131542	-2.151873

C	-4.873995	5.928332	-4.530111
C	-5.125394	4.293283	-1.119741
H	-3.999736	5.905260	-1.968348
H	-4.683084	4.423889	-0.135483
H	-5.376968	5.685169	-5.469868
H	-3.793136	5.878668	-4.702480
H	-5.107707	6.970415	-4.284737
C	-6.064408	2.394138	2.326202
C	-6.269678	2.992015	3.564495
H	-5.514942	1.458148	2.278614
C	-6.971526	4.185428	3.682584
H	-5.871678	2.518531	4.458750
C	-7.466634	4.767159	2.514609
C	-7.202958	4.833045	5.011330
C	-7.260425	4.178324	1.279771
H	-8.021290	5.700652	2.577934
H	-7.646094	4.667628	0.388749
H	-6.675931	4.307721	5.812432
H	-8.269171	4.845441	5.265007
H	-6.866524	5.875476	5.010352
C	-2.224874	-2.709846	-2.713558
C	-2.337176	-3.312871	-3.960721
H	-1.480606	-1.931289	-2.566974
C	-3.276592	-4.309945	-4.196799
H	-1.676753	-3.001576	-4.766407
C	-4.106643	-4.685247	-3.139464
C	-3.402251	-4.970911	-5.533374
C	-3.997233	-4.089348	-1.895247
H	-4.850850	-5.462584	-3.298142
H	-4.654339	-4.410514	-1.091343
H	-2.685324	-4.564144	-6.251779
H	-4.407260	-4.839466	-5.949632
H	-3.229392	-6.050484	-5.460608

C	-3.940426	-2.964364	1.995229
C	-4.014002	-3.806286	3.098879
H	-4.480021	-2.021806	2.014887
C	-3.335916	-5.018967	3.128535
H	-4.614023	-3.511292	3.956348
C	-2.575850	-5.367190	2.010959
C	-3.402411	-5.928393	4.314708
C	-2.503929	-4.534702	0.908484
H	-2.031885	-6.309070	2.008439
H	-1.912180	-4.841339	0.049378
H	-4.100098	-5.554419	5.068968
H	-2.419818	-6.033193	4.788796
H	-3.722570	-6.935736	4.026715
C	1.785238	-1.019622	0.447481
C	2.089043	-2.376594	0.337211
H	2.554879	-0.276052	0.622860
S	0.629523	-3.343389	0.051571
C	-11.202456	0.957494	-1.006776
C	-11.507174	2.313986	-0.893763
H	-11.970701	0.214663	-1.191125
S	-10.051881	3.278373	-0.579071
C	-12.851292	2.733924	-1.006431
C	-13.490689	3.943979	-0.931113
H	-13.484630	1.871541	-1.192366
C	-12.840038	5.247919	-0.713025
C	-14.923020	4.176630	-1.053330
C	-15.144847	5.630139	-0.906707
C	-13.910954	6.257635	-0.708731
C	-16.300540	6.405473	-0.928502
C	-16.181658	7.779856	-0.755303
H	-17.283590	5.977561	-1.073316
C	-14.944366	8.388275	-0.563298
H	-17.078356	8.390515	-0.769542

C	-13.787219	7.621492	-0.537529
H	-14.888580	9.463898	-0.433269
H	-12.807841	8.065382	-0.388245
O	-11.654662	5.467884	-0.566932
C	-15.924463	3.254456	-1.259224
C	-15.714348	1.855450	-1.392284
C	-17.298992	3.599464	-1.363928
N	-15.583940	0.712657	-1.504310
N	-18.427822	3.827787	-1.456661
C	3.432084	-2.798946	0.456505
C	4.067850	-4.012019	0.398137
H	4.068277	-1.935792	0.628909
C	3.415873	-5.314223	0.172989
C	5.498410	-4.249070	0.533515
C	5.719057	-5.701471	0.374257
C	4.484980	-6.325749	0.167957
C	4.359631	-7.688310	-0.012252
C	5.515509	-8.457041	0.012079
H	3.380045	-8.129860	-0.166965
C	6.753230	-7.851632	0.210799
H	5.458485	-9.531771	-0.124647
C	6.873615	-6.478660	0.393658
H	7.649105	-8.463550	0.222959
H	7.856983	-6.052909	0.542782
C	6.498750	-3.332932	0.769042
C	6.289299	-1.937031	0.931663
C	7.871266	-3.682580	0.884782
N	6.159470	-0.796975	1.069200
N	8.998471	-3.914648	0.987402
O	2.231146	-5.531427	0.018062

Optimized Geometry of IDTBR by M06/6-311G**

92

Coordinates from ORCA-job OIDTBR-opt

N	3.45364845440313	-12.81285238358722	1.80452997879137
C	4.35857922418802	-11.92001811603504	1.32369387349397
C	3.95245831247935	-14.01711457276918	2.32556373852805
S	6.00017055757327	-12.56217293321390	1.47611531346596
C	5.42184570788005	-14.05189058406386	2.20604975301415
C	6.10372507982317	-15.13378510071163	2.63095707799640
C	9.87715334344299	-14.89893745399560	2.26067387298654
C	10.35081286224672	-16.12227405513475	2.69536868359415
C	8.51547167075785	-14.55597634022387	2.23341369018788
C	9.34751535040594	-17.05224176727169	3.13613139482557
C	7.51092812768978	-15.41440879972099	2.63500432502534
C	7.95027420323545	-16.69790267581338	3.10358876624117
O	3.24812409838754	-14.87623641163336	2.79297271133853
C	2.01552056020674	-12.54051292394414	1.85811081732809
C	1.64134984343842	-11.81693997066807	3.13158845846783
N	9.56208383382185	-18.27325851779781	3.61425623833715
S	8.11097238211182	-18.90872461165459	3.98041777217114
N	7.15585349863182	-17.66308253585863	3.55041792174332
C	11.75591174427762	-16.45026135045980	2.71186599340515
S	12.95606727236496	-15.15778446732480	2.62495967676475
C	12.36143229692349	-17.69081971864841	2.76169439896375
C	14.22631338088078	-16.31556217159691	2.64367058866067
C	13.75866088223796	-17.60533469341137	2.71425572467663
C	15.66870926783675	-16.28954374602399	2.61554098369328
C	16.09028073773960	-17.63772781700863	2.66107769246583
C	14.90276836370075	-18.58582808312465	2.71704033592390
C	16.58277831213263	-15.23699292520114	2.55736314843029
C	17.92619451393929	-15.55450285679309	2.54462365912050
C	17.43296152935756	-17.95553354808094	2.64430197205074
C	18.34623862437243	-16.90293301285178	2.58550594950695

C	19.11679837774981	-14.60827340658192	2.49992795692588
C	20.25967704193159	-15.59144050557059	2.50698322047303
C	19.78734940827803	-16.88005209829966	2.55965347319784
C	21.65736273686493	-15.51218081092207	2.47695377188528
C	22.25974367116506	-16.75644475114795	2.51113083359183
S	21.04960506676447	-18.04439229476237	2.57261954120144
C	23.66169082958706	-17.09583436499320	2.51764181439490
C	24.69025685274197	-16.11528310555126	2.29390118399941
C	24.11508925973294	-18.38386444199382	2.73936975331432
C	26.08255182774197	-16.49154783180090	2.30841348040897
C	25.47090102334881	-18.74617572759398	2.75094445458929
C	26.49668812335941	-17.84499238573929	2.54367285483096
N	24.50803009577958	-14.82190298435427	2.05072181334621
S	25.97839729749971	-14.15152754874497	1.86527802592270
N	26.90160312622202	-15.47311577260585	2.07948510797280
C	27.90038235917722	-18.13821497095227	2.54672723205282
S	27.93646900254179	-20.93202524460205	2.92487092876397
C	28.55763967199591	-19.30584769624259	2.69123677461071
C	29.56594696814994	-21.62120730398867	2.95946153053605
C	30.03055242110023	-19.32954819738849	2.64480249906817
N	30.49833399777431	-20.64260281994121	2.81279789188194
O	30.76007982287846	-18.38285457668452	2.48714267654166
C	31.93763428394378	-20.90816019703289	2.75458529863387
C	32.38979958050227	-21.18012153047096	1.33802880430552
C	14.91274316202419	-19.43104116871802	3.98891750881650
H	15.78485657927075	-20.09398811480165	4.00586056593439
C	14.86230861523887	-19.48628883405116	1.48269871717775
H	13.96125807904112	-20.11011035429552	1.49261574680175
S	29.81214625438263	-23.23657310872311	3.15223737943514
S	4.07022575278135	-10.43730393948075	0.67202728339618
C	19.15079232207668	-13.71013329297896	3.73586966456146
H	20.05250158302985	-13.08712535731881	3.73131544079515
C	19.11698334109073	-13.76011106411615	1.22975791132518

H	18.24583115050397	-13.09594900017544	1.20889586125784
H	5.46820920816005	-15.92208828516130	3.03382147616629
H	28.55758037268642	-17.28097653950836	2.40362338504350
H	10.58209197875514	-14.15735907898622	1.89700545609969
H	8.27565919072951	-13.56194212299386	1.87283613080628
H	1.75960100383736	-11.95553888172527	0.97191073529445
H	1.51859116032737	-13.51043335952798	1.79055422657693
H	2.15698580656593	-10.85454065830379	3.20290864298180
H	0.56478246401064	-11.62745371136050	3.15303256399123
H	1.89844173374969	-12.41526906542635	4.01109379474124
H	11.79144099995154	-18.61033974611323	2.81447384181049
H	16.24660576686274	-14.20277636539518	2.52667476212445
H	17.76961114734049	-18.98953935809412	2.67574218807871
H	22.23342015752886	-14.59602934455101	2.43944207759745
H	23.39554320649635	-19.17508947664025	2.92597464481433
H	25.68907899969490	-19.78918535963489	2.95056872214542
H	32.42773449085178	-20.02212448363940	3.16387973248363
H	32.14147447694280	-21.75382156073535	3.41464289197891
H	32.17759032165934	-20.32377494838261	0.69075786240831
H	33.46741961970326	-21.36344108473751	1.31656966808029
H	31.88736016656120	-22.05994075758609	0.92480943190917
H	14.93953089501187	-18.80155385874732	4.88468000287293
H	14.01320680611827	-20.05559707580447	4.03463534626455
H	14.85963019000619	-18.89311459685537	0.56199240668549
H	15.73374165404710	-20.15011683661519	1.46243404347124
H	18.28004360274251	-13.04528553501958	3.75253792656658
H	19.14832430749182	-14.30442261778718	4.65582327914787
H	20.01760871549022	-13.13661313456983	1.19116396839725
H	19.09486783391586	-14.38747320884773	0.33237927831128

To achieve a satisfactory agreement between simulated and experimental spectra, we saw that the increasing participation of higher-energy virtual states with increasing core-electron binding energy was needed. NEXAFS spectra were simulated for the C K-edge using 1000 roots and the lowest 20 unoccupied orbitals to cover only the π* region. Particularly for ITIC, the number of unoccupied orbitals used was 40 to ensure that all transitions in this region were described. In N K-edge simulations, 500 roots and the lowest 60 unoccupied orbitals were used. For the O K-edge, 500 roots and 120 unoccupied orbitals were included to ensure an adequate description of both π* and a partial description of σ* regions. Even though the description of the σ* region is not our priority, in the case of oxygen, its calculation becomes more relevant so that we can separate the π* region properly, since there is no clear ionization potential step in the spectra to separates the two regions, as is the case in the C K-edge. Furthermore, in the experimental O K-edge spectrum we observe very clear peaks referring to σ* transitions, which is not the case for the N K-edge for example, then, also becoming another reason for the need for at least a partial description.

The assignment between theoretical and experimental features was performed after aligning the energy of the first calculated transition with the first experimental peak maximum. The remaining assignments were then based on two complementary criteria: i) energy compatibility between the calculated and experimental features and ii) consistency between the number of relevant peaks, the relative intensities in the angular dependence simulation, and the final state character of the transitions.

The vectors employed in the simulated field are unit vectors located in the first quadrant, since only their direction is physically relevant and not their magnitude. Consequently, all vector components have values between 0 and 1, and, as a result, the oscillator strengths obtained after applying the angular correction are reduced in some orders of magnitude. In order to display both spectra on the same graph and facilitate visual comparison, it was necessary to apply a normalization factor in the intensity of the gaussian spectrum calculated without angular dependence. Our choice was to scale the spectra such that the non-angular spectrum approximately outlines the angular-dependent spectra to provide a clearer visualization of the relative intensity variations. This normalization factor was different for each molecule and absorption edge because the transition dipole distributions are inherently different in each case, and their values were choose solely by visual inspection. The Python script uses the TDDFT output file directly as an input, and after each data treatment step we checked the data frame to ensure that all data were corrected applied.

In many cases, especially for the carbon spectrum, the experimental peaks arise from a convolution of multiple transitions. When the set of transitions contributing to a given peak exhibits very similar intensities, the transition that best represents the peak was selected based on the angular dependence of the oscillator strength, such that the vertical transition most affected by angular variation was considered the most representative. When the responses to angular variations were equally significant, we chose to report the full set of transitions.

2- Complementary figures

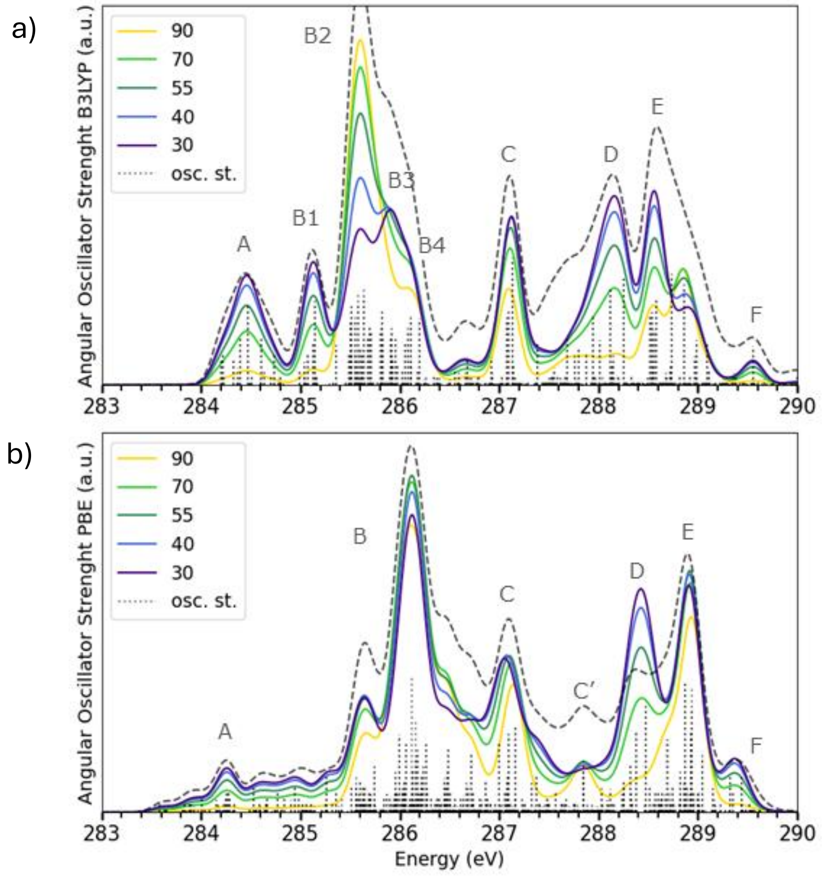

Figure S2. Calculated carbon K-edge NEXAFS obtained from TD-DFT calculation at (a) B3LYP/6-311G**, and (b) PBE/6-311G** theory level for ITIC.

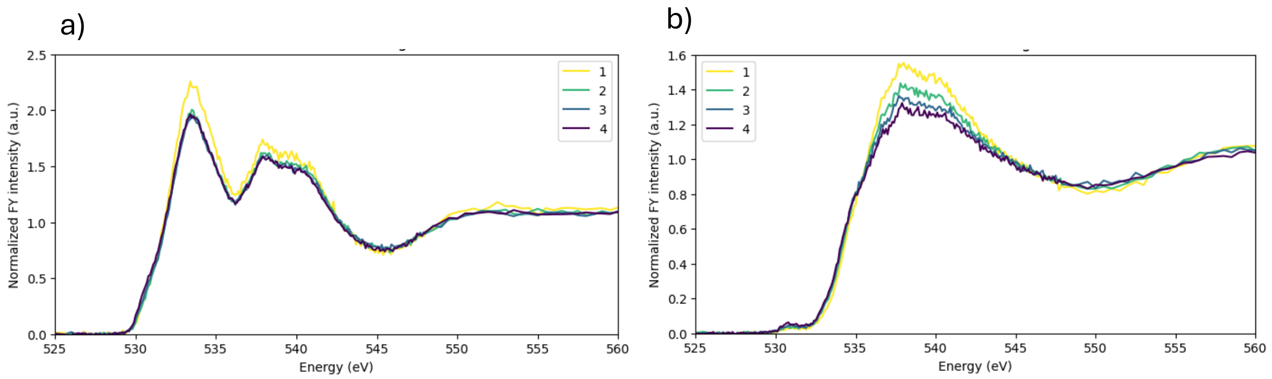

Figure S3. Oxygen K-edge NEXAFS in FY mode of (a) ITIC and (b) IDTBR.

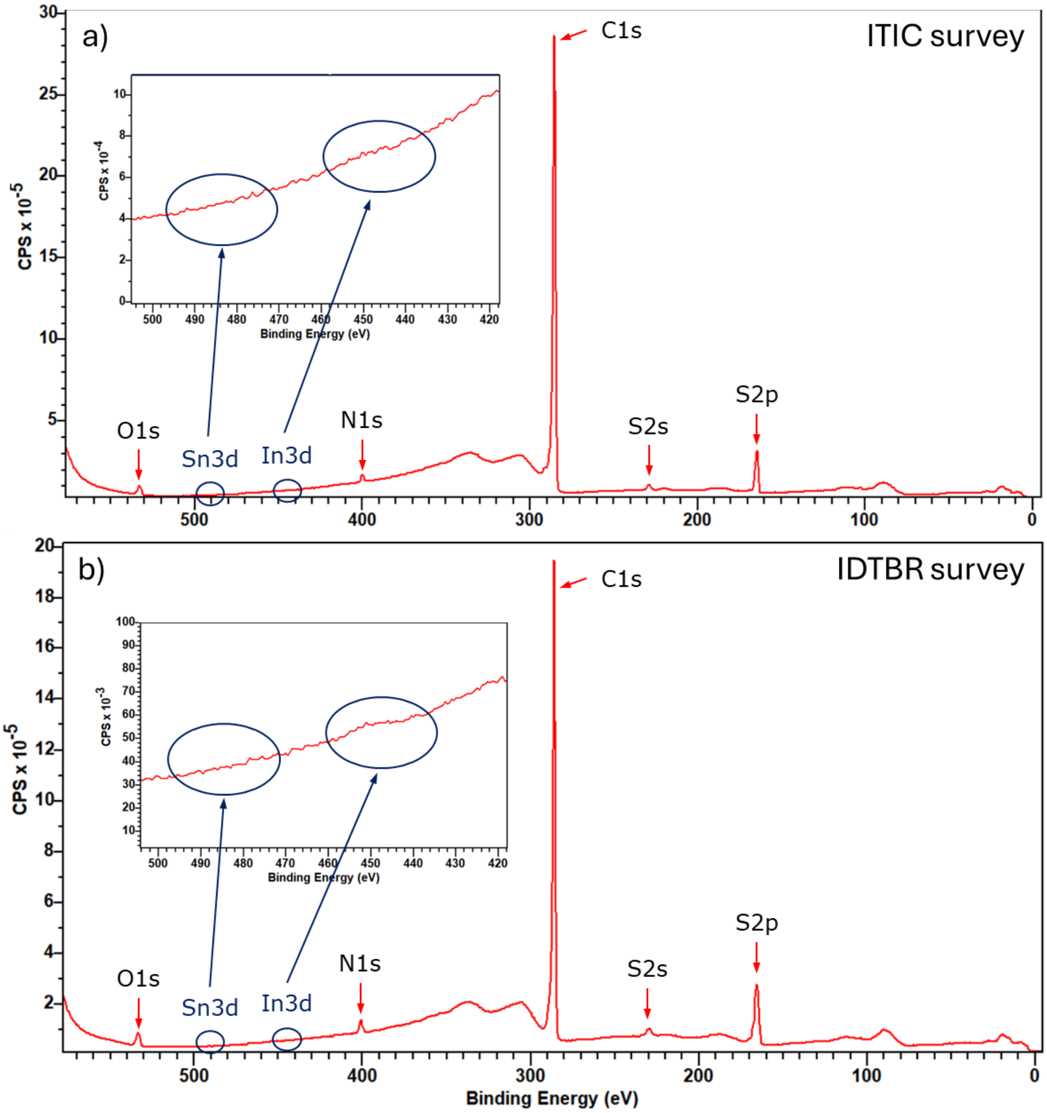

Figure S4. XPS survey spectra from 0 to 600 eV for (a) ITIC and (b) IDTBR. The most intense signal for indium and tin is the 3d5/2-3/2 peak, around 443-448 eV and 485-490 eV, respectively. The only significant signals in both spectra come only from sulfur, carbon, nitrogen, and oxygen present in the samples.

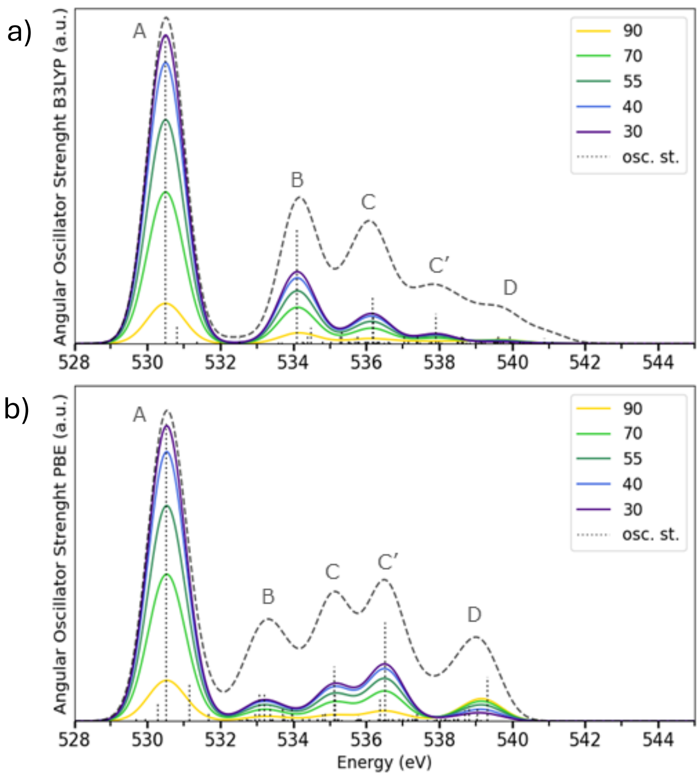

Figure S5. Calculated oxygen K-edge NEXAFS obtained from TD-DFT calculation at (a) B3LYP/6-311G**, and (b) PBE/6-311G** theory level for ITIC.

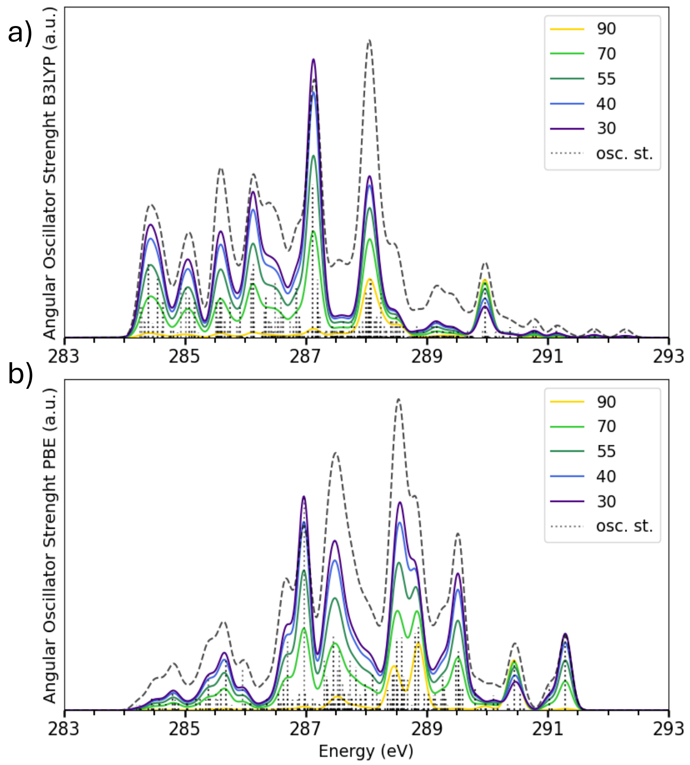

Figure S6. Calculated carbon K-edge NEXAFS obtained from TD-DFT calculation at (a) B3LYP/6-311G**, and (b) PBE/6-311G** theory level for IDTBR.

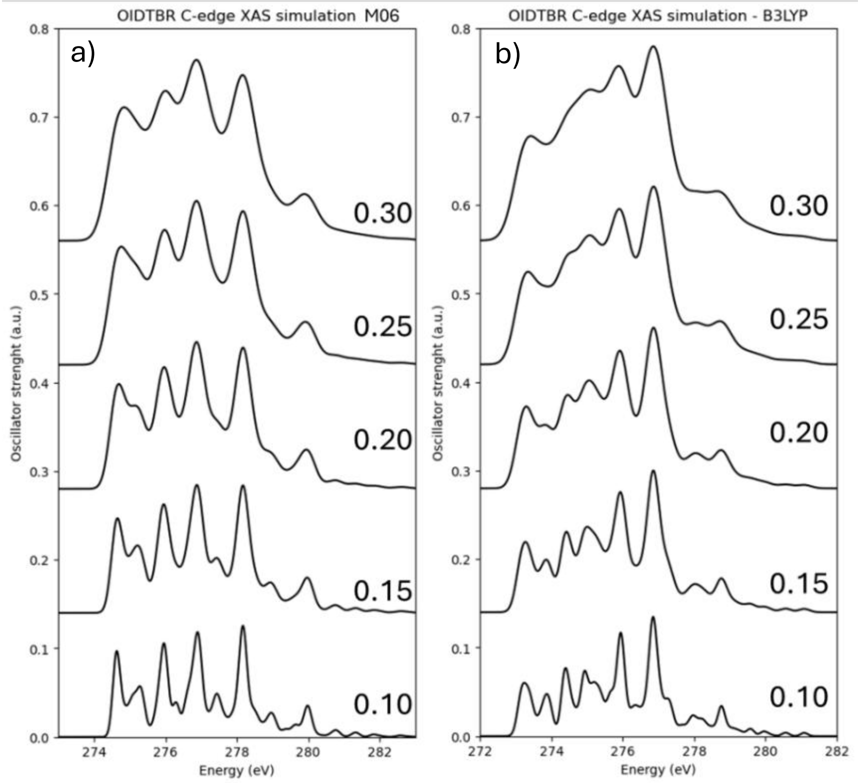

Figure S7. Calculated carbon K-edge NEXAFS for different values of gaussian broadening obtained from TD-DFT calculation at (a) M06/6-311G**, and (b) B3LYP/6-311G** theory level for IDTBR. It's interesting to note that the best spectral broadening for this set, i.e., the one that most resembles the experimental spectrum, should be something around 0.20-0.25. But, since we choose to maintain the same value for a specific edge independently of the molecule, the value 0.10 was the best to reproduce both ITIC and IDTBR experimental spectra.

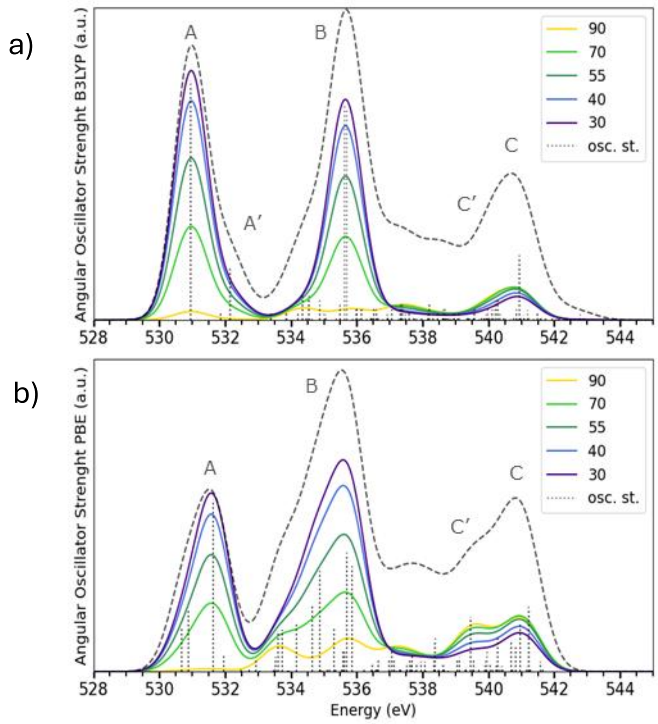

Figure S8. Calculated oxygen K-edge NEXAFS obtained from TD-DFT calculation at (a) B3LYP/6-311G**, and (b) PBE/6-311G** theory level for IDTBR.

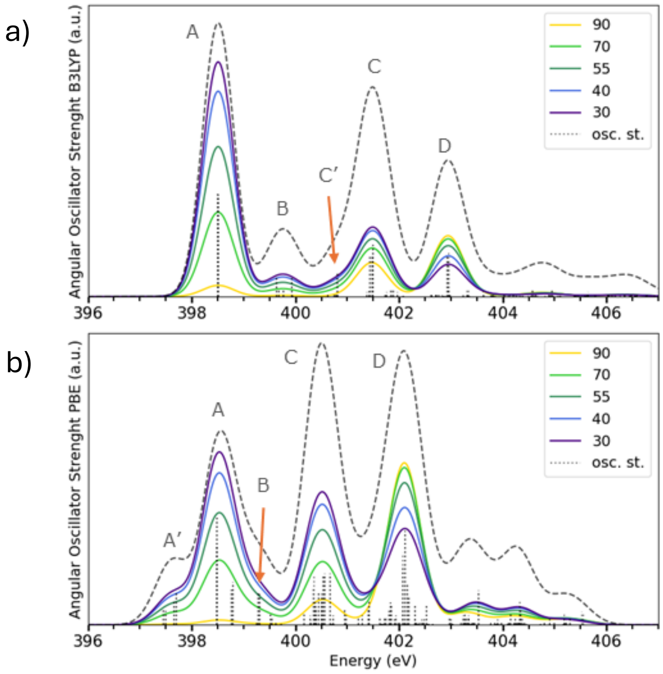

Figure S9. Calculated nitrogen K-edge NEXAFS obtained from TD-DFT calculation at (a) B3LYP/6-311G**, and (b) PBE/6-311G** theory level for IDTBR.

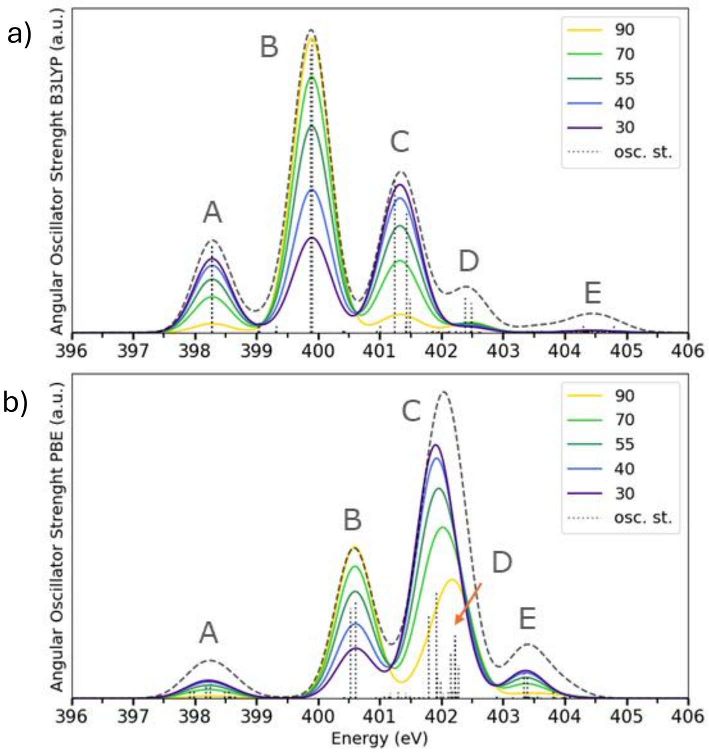

Figure S10. Calculated nitrogen K-edge NEXAFS obtained from TD-DFT calculation at (a) B3LYP/6-311G**, and (b) PBE/6-311G** theory level for ITIC.
